# Targeting Progesterone Receptor Membrane Component 1 to Improve Muscle Development and Glucose Homeostasis

**DOI:** 10.1002/jcsm.70121

**Published:** 2025-11-10

**Authors:** Sang R. Lee, Moeka Mukae, Globinna Kim, Jung‐Eun Park, Young Hoon Sung, Young Suk Won, Tae Won Kim, Hyo‐Jung Kwun, In‐Jeoung Baek, Eui‐Ju Hong

**Affiliations:** ^1^ College of Veterinary Medicine Chungnam National University Daejeon Republic of Korea; ^2^ Department of Physiology Dong‐A University College of Medicine Busan Republic of Korea; ^3^ Department of Translational Biomedical Sciences, Graduate School of Dong‐A University Busan Republic of Korea; ^4^ Department of Cell and Genetic Engineering University of Ulsan College of Medicine, Asan Medical Center Seoul Republic of Korea; ^5^ Laboratory Animal Resource and Research Center Korea Research Institute of Bioscience and Biotechnology Cheongju Republic of Korea

**Keywords:** glucose homeostasis, insulin resistance, PGRMC1, skeletal muscle, type 2 diabetes

## Abstract

**Background:**

Type 2 diabetes mellitus (T2D) arises from the interplay between peripheral insulin resistance and pancreatic β‐cell dysfunction, ultimately leading to impaired glucose utilization and chronic hyperglycemia. Despite therapeutic advances, the multifactorial nature of T2D continues to demand the development of novel treatment strategies. Progesterone receptor membrane component 1 (PGRMC1) has emerged as a potential modulator of metabolic function, though its role in T2D pathogenesis has not been fully elucidated.

**Methods:**

To investigate the role of PGRMC1 in T2D, we generated skeletal muscle‐specific *Pgrmc1* knockout (PKO) mice (*ACTA* ^cre^‐*Pgrmc1* ^fl/fl^). T2D was induced via a high‐fat diet combined with streptozotocin (HFD‐STZ) or using genetically diabetic (*lepr* ^db^/*lepr* ^db^; *db/db*) mice. A small‐molecule screen of 330 compounds identified 11α‐hydroxyprogesterone (11α‐OHP) as a PGRMC1‐modulating candidate. The antidiabetic efficacy of 11α‐OHP was assessed in vitro and across multiple in vivo diabetic models. Whole‐body PKO mice were used to evaluate the systemic consequences of global Pgrmc1 deletion. Glucose tolerance test (GTT), insulin tolerance test (ITT) and modified homeostatic model assessment for insulin resistance (HOMA‐IR, 5‐h fasting) were used to evaluate glucose metabolism. Real‐time cell metabolism analyser (Seahorse analysis) was used for measuring cellular glycolysis.

**Results:**

Skeletal muscle PKO improved glucose clearance in GTT (*p* < 0.0001) and insulin sensitivity in ITT (*p* < 0.0001). Skeletal muscle PKO mice under T2D suppressed insulin resistance according to reduced modified HOMA‐IR (*p* < 0.05) and promoted muscle development (quadriceps femoris, gastrocnemius, tibialis anterior muscle and extensor digitorum longus; *p* < 0.05). Mechanistically, PGRMC1 interacted with PPP2R5D, a PP2A regulatory subunit, which dephosphorylates RSK1. PGRMC1 loss suppressed PP2A activity, increasing RSK1 phosphorylation and activating AKT signalling, thereby enhancing myoblast proliferation (*p* < 0.05), differentiation (*p* < 0.01) and glycolysis (*p* < 0.0001). 11α‐OHP facilitated proteasomal degradation of PGRMC1, elevated pAKT levels and improved glucose clearance in GTT (*p* < 0.0001) and insulin sensitivity in ITT (*p* < 0.0001) in wild‐type mice but not in PKO mice. Notably, 11α‐OHP restored glucose clearance in GTT (*p* < 0.0001) and insulin sensitivity in ITT (*p* < 0.0001) and increased muscle mass in both HFD‐STZ and *db/db* mice, but its effects were abolished in skeletal muscle PKO mice. Whole‐body PKO mice still increased muscle development and metabolic activation, suggesting minimal interference by systemic PKO.

**Conclusions:**

These findings identify skeletal muscle *PGRMC1* as a pivotal regulator of glucose metabolism and highlight its inhibition as a promising muscle‐targeted therapeutic approach for T2D management.

## 1 Introduction

Type 2 diabetes mellitus (T2D) is a rapidly growing global health burden, with its prevalence projected to reach 12.2% (783.2 million) by 2045 [1]. Despite the availability of pharmacological and lifestyle interventions, a significant subset of patients remains unresponsive to current therapies, highlighting the urgent need for novel therapeutic strategies [2].

Skeletal muscle plays a pivotal role in glucose homeostasis, as insulin promotes glucose uptake in skeletal muscle while suppressing hepatic gluconeogenesis [3]. Approximately 25% of whole‐body glucose utilization in the postabsorptive state occurs via skeletal muscle [4], making it a primary regulator of systemic glycemia [5]. T2D development is closely linked to skeletal muscle dysfunction, which can be categorized into two primary mechanisms: (1) reduced muscle mass and (2) impaired muscle metabolism. First, impaired glucose clearance due to decreased skeletal muscle mass itself has been directly associated with T2D development [6, 7]. Second, metabolic dysfunction within skeletal muscle contributes to insulin resistance. Insulin resistance initially manifests in skeletal muscle, driven by adipokines from adipose tissue [8, 9], and elevated plasma‐free fatty acids [10], eventually progressing to adipose tissue and the liver [11]. These findings suggest that both maintaining skeletal muscle mass and enhancing skeletal muscle metabolism could serve as effective therapeutic approaches for T2D. Clinically, T2D patients exhibit a significant reduction in metabolic enzyme activity within skeletal muscle [12], supporting the idea that skeletal muscle is a key therapeutic target. Consequently, numerous strategies have been explored to enhance muscle glucose uptake, microvascular function and mitochondrial metabolism [13, 14, 15].

Progesterone receptor membrane component 1 (Pgrmc1) is a noncanonical progesterone receptor involved in various non‐genomic metabolic processes [16, 17, 18, 19]. Previous studies have associated Pgrmc1 with pancreatic glucagon‐like peptide‐1 (GLP‐1) signalling, which promotes insulin secretion [20], and with direct interactions with the insulin receptor [21]. Pgrmc1 has also been shown to regulate glucose and lipid uptake in adipocytes [22]. Because skeletal muscle is the principal site of glucose disposal, clarifying the contribution of muscle Pgrmc1 to systemic glucose metabolism is critical. Here, we investigated the role of Pgrmc1 in whole‐body glucose homeostasis using skeletal muscle–specific Pgrmc1 knockout (PKO) mice (*ACTA* ^cre^‐*Pgrmc1* ^fl/fl^). We found that loss of Pgrmc1 in skeletal muscle markedly enhanced glucose clearance and insulin sensitivity, supporting Pgrmc1 as a potential therapeutic target in T2D. Finally, we tested a Pgrmc1‐lowering compound, which improved glucose metabolism during both the development and recovery phases of T2D.

## 2 Materials and Methods

### 2.1 Animals

C57BL/6J male mice were housed in a pathogen‐free facility at Chungnam National University under a standard 12‐h light/dark cycle and provided either standard chow or a high‐fat diet along with water. *Db/db* male mice (*lepr* ^db^/*lepr* ^db^) were obtained from Jackson Laboratory. The high‐fat diet (#D12492, Research Diets Inc., New Brunswick, NJ) consisted of 20% kcal from carbohydrates, 20% kcal from protein and 60% kcal from fat. All mouse experiments were conducted in accordance with the guidelines of the Chungnam Facility Animal Care Committee (approval number: 202006A‐CNU‐105). All mice used for the experiments were around 8 weeks of age when subjected to experimental procedures. T2D was induced by administering streptozotocin (30 mg/kg, intraperitoneally) in combination with a high‐fat diet at designated time points, following previously established protocols [23]. For acute Pgrmc1‐lowering drug administration, 11α‐hydroxyprogesterone (11α‐OHP) was dissolved in a mixture of PBS and ethanol and injected intraperitoneally for two consecutive days. For long‐term administration, 11α‐OHP was dissolved in a mixture of corn oil and ethanol and injected subcutaneously twice weekly. All control mice received the same dose and composition of the respective solvent. To assess skeletal muscle proportions, individual muscles were carefully dissected from their insertion points to their endpoints. Epididymal adipose tissue was isolated for adipose tissue analysis. Among the T2D mice, some underwent dual‐energy X‐ray absorptiometry (DEXA) to evaluate whole‐body lean and fat mass.

### 2.2 Additional Methods

Additional materials and methods are provided in the Supporting Information.

### 2.3 Statistics

Data are presented as mean ± SD. Statistical differences between groups were assessed using Student's *t*‐test or one‐way ANOVA followed by Tukey's multiple comparisons test or two‐way ANOVA (column factor) that was performed. All statistical analyses were performed using GraphPad Software (GraphPad Inc., San Diego, CA).

## 3 Results

### 3.1 Skeletal Muscle‐Specific *Pgrmc1* Knockout Enhances Skeletal Muscle Development and Improves Glucose Clearance and Insulin Sensitivity

**FIGURE 1:**
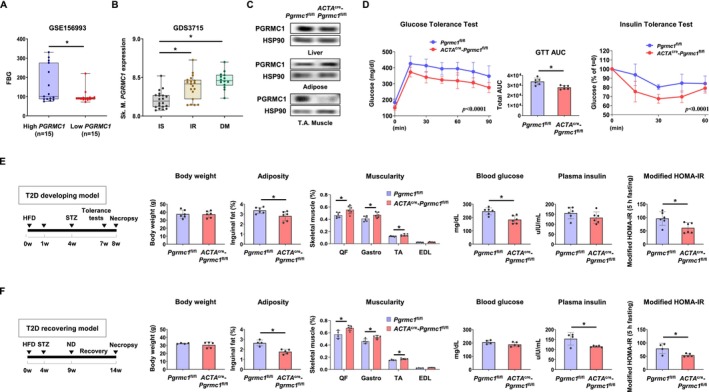
Skeletal muscle‐specific PKO mice exhibit enhanced insulin sensitivity and muscle development during T2D progression and recovery. (A) GEO dataset (GSE156993, exemplar) showing fasting blood glucose (FBG) levels in patients with high or low PGRMC1 mRNA expression in peripheral blood mononuclear cells (PBMCs). Each group consisted of 15 patients. (B) GEO dataset (GDS3715) presenting PGRMC1 mRNA expression levels in skeletal muscle (Sk. M.) of patients classified as insulin‐sensitive (IS, 20 of 20), insulin‐resistant (IR, 19 of 20), and diabetes mellitus (DM, 14 of 15) conditions. (C) Western blot analysis and quantification of PGRMC1 expression in liver, adipose tissue, and skeletal muscle of *Pgrmc1* ^fl/fl^ and *ACTA* ^cre^‐*Pgrmc1* ^fl/fl^ mice. HSP90 was used as an internal control. (D) Glucose tolerance test (GTT) and insulin tolerance test (ITT) of *Pgrmc1* ^fl/fl^ and *ACTA* ^cre^‐*Pgrmc1* ^fl/fl^ mice. The area under the curve (AUC) for GTT is presented. (E, F) Experimental schedules for T2D development or recovery in *Pgrmc1* ^fl/fl^ and *ACTA* ^cre^‐*Pgrmc1* ^fl/fl^ mice. Streptozotocin (STZ, 30 mg/kg) was administered intraperitoneally. Body weight (BW, g) and inguinal adipose tissue weight per BW (adiposity, %) of *Pgrmc1* ^fl/fl^ and *ACTA* ^cre^‐*Pgrmc1* ^fl/fl^ mice. Individual skeletal muscle weights in the hindlimb (quadriceps femoris, QF; gastrocnemius, Gastro; tibialis anterior, TA; extensor digitorum longus, EDL) normalized to BW (muscularity, %) in *Pgrmc1* ^fl/fl^ and *ACTA* ^cre^‐*Pgrmc1* ^fl/fl^ mice. Blood glucose (mg/dL) and plasma insulin (uIU/mL) levels of *Pgrmc1* ^fl/fl^ and *ACTA* ^cre^‐*Pgrmc1* ^fl/fl^ under fasted conditions (5 h). Modified HOMA‐IR values (blood glucose x plasma insulin/405) representing insulin resistance in *Pgrmc1* ^fl/fl^ and *ACTA* ^cre^‐*Pgrmc1* ^fl/fl^ mice under fasting conditions (5 h, modified for resting state simulation). Mice used for the experiment: 6 for T2D‐developing *Pgrmc1* ^fl/fl^, 6 for T2D‐developing *ACTA* ^cre^‐*Pgrmc1* ^fl/fl^, 4 for T2D‐recovering *Pgrmc1* ^fl/fl^ and 5 for T2D‐recovering *ACTA* ^cre^‐*Pgrmc1* ^fl/fl^. Values represent mean ± SD *p < 0.05, Student's *t*‐test or one‐way ANOVA followed by Tukey's multiple comparisons test. Two‐way ANOVA (column factor) was performed for GTT and ITT.

To determine the clinical relevance of *PGRMC1* in glucose metabolism, we first analysed human cohort data. Interestingly, patients with lower *PGRMC1* expression in peripheral blood mononuclear cells exhibited reduced fasting blood glucose levels compared to those with higher *PGRMC1 expression* (Figure 1A). Consistently, patients with insulin resistance (IR) or diabetes mellitus (DM) exhibited higher *PGRMC1* expression in skeletal muscle compared to those with insulin sensitivity (IS) (Figure 1B). To investigate the biological significance of *Pgrmc1* in skeletal muscle and its role in regulating whole‐body glucose levels, *Pgrmc1* floxed (*Pgrmc1* ^fl/fl^) mice were crossed with *ACTA* ^cre^ mice to generate a skeletal muscle‐specific PKO (*ACTA* ^cre^‐*Pgrmc1* ^fl/fl^) model (Figure S1). To validate the model, PGRMC1 protein levels were assessed in the liver, adipose tissue and skeletal muscle of *Pgrmc1* ^fl/fl^ and *ACTA* ^cre^‐*Pgrmc1* ^fl/fl^ mice. PGRMC1 was present in the liver and adipose tissue of both models; however, it was absent in the skeletal muscle of *ACTA* ^cre^‐*Pgrmc1* ^fl/fl^ mice while still expressed in *Pgrmc1* ^fl/fl^ mice (Figure 1C). *ACTA* ^cre^‐*Pgrmc1* ^fl/fl^ mice showed a significant reduction in blood glucose levels in both GTT and ITT compared to *Pgrmc1* ^fl/fl^ mice (Figure 1D), suggesting improved glucose metabolism and insulin sensitivity.

To determine the role of skeletal *Pgrmc1* in glucose homeostasis under T2D conditions, mice were subjected to either a T2D development or a subsequent recovery phase. Specifically, mice were fed a HFD for 8 weeks, and a low dose of streptozotocin (30 mg/kg) was administered at week 4 of HFD feeding to induce T2D. For the recovery phase, after 9 weeks of T2D induction, mice were switched to a normal chow diet for 5 weeks to facilitate metabolic rehabilitation. This model provides preclinical evidence that Pgrmc1‐mediated pathways may contribute to the reversibility of T2D progression through lifestyle modification without pharmacological intervention. In the T2D development phase, *ACTA* ^cre^‐*Pgrmc1* ^fl/fl^ mice exhibited similar body weight to *Pgrmc1* ^fl/fl^ mice but showed decreased adiposity (Figure 1E). The lean/body mass ratio (muscularity) was significantly increased in *ACTA* ^cre^‐*Pgrmc1* ^fl/fl^ mice compared to *Pgrmc1* ^fl/fl^ mice (Figure 1E). Despite unchanged plasma insulin levels, blood glucose levels were lower in *ACTA* ^cre^‐*Pgrmc1* ^fl/fl^ mice (Figure 1E). Accordingly, *ACTA* ^cre^‐*Pgrmc1* ^fl/fl^ mice exhibited lower modified HOMA‐IR levels, suggesting that skeletal PGRMC1 depletion enhances whole‐body insulin sensitivity under metabolic stress (Figure 1E). Given that PGRMC1 remains expressed in other organs, the decrease of blood glucose level further indicates heightened insulin sensitivity specifically in skeletal muscle. In the recovery phase, similar trends were observed. Despite having comparable body weight to *Pgrmc1* ^fl/fl^ mice, *ACTA* ^cre^‐*Pgrmc1* ^fl/fl^ mice maintained lower adiposity and a significantly higher muscularity (Figure 1F), consistent with the findings from the T2D development phase. Despite the blood glucose levels being similar, the plasma insulin levels were significantly lower in *ACTA* ^cre^‐*Pgrmc1* ^fl/fl^ mice compared to *Pgrmc1* ^fl/fl^ mice (Figure 1F). Since blood glucose levels returned to normal after T2D recovery in *Pgrmc1* ^fl/fl^ mice but remained stable throughout in *ACTA* ^cre^‐*Pgrmc1* ^fl/fl^ mice, this suggests that their blood glucose levels were already well maintained during T2D development. Instead, the marked reduction in plasma insulin levels after recovery implies that less insulin is required to maintain glucose homeostasis within the normal physiological range in *ACTA* ^cre^‐*Pgrmc1* ^fl/fl^ mice. Concordantly, modified HOMA‐IR levels were significantly lower in *ACTA* ^cre^‐*Pgrmc1* ^fl/fl^ mice than in *Pgrmc1* ^fl/fl^ mice (Figure 1F). Together, these findings indicate that skeletal PGRMC1 depletion enhances insulin sensitivity during both T2D development and recovery phases.

### 3.2 PGRMC1 Regulates Cellular Development and Glycolysis in Skeletal Muscle via the PP2A‐RSK1‐AKT Axis

**FIGURE 2:**
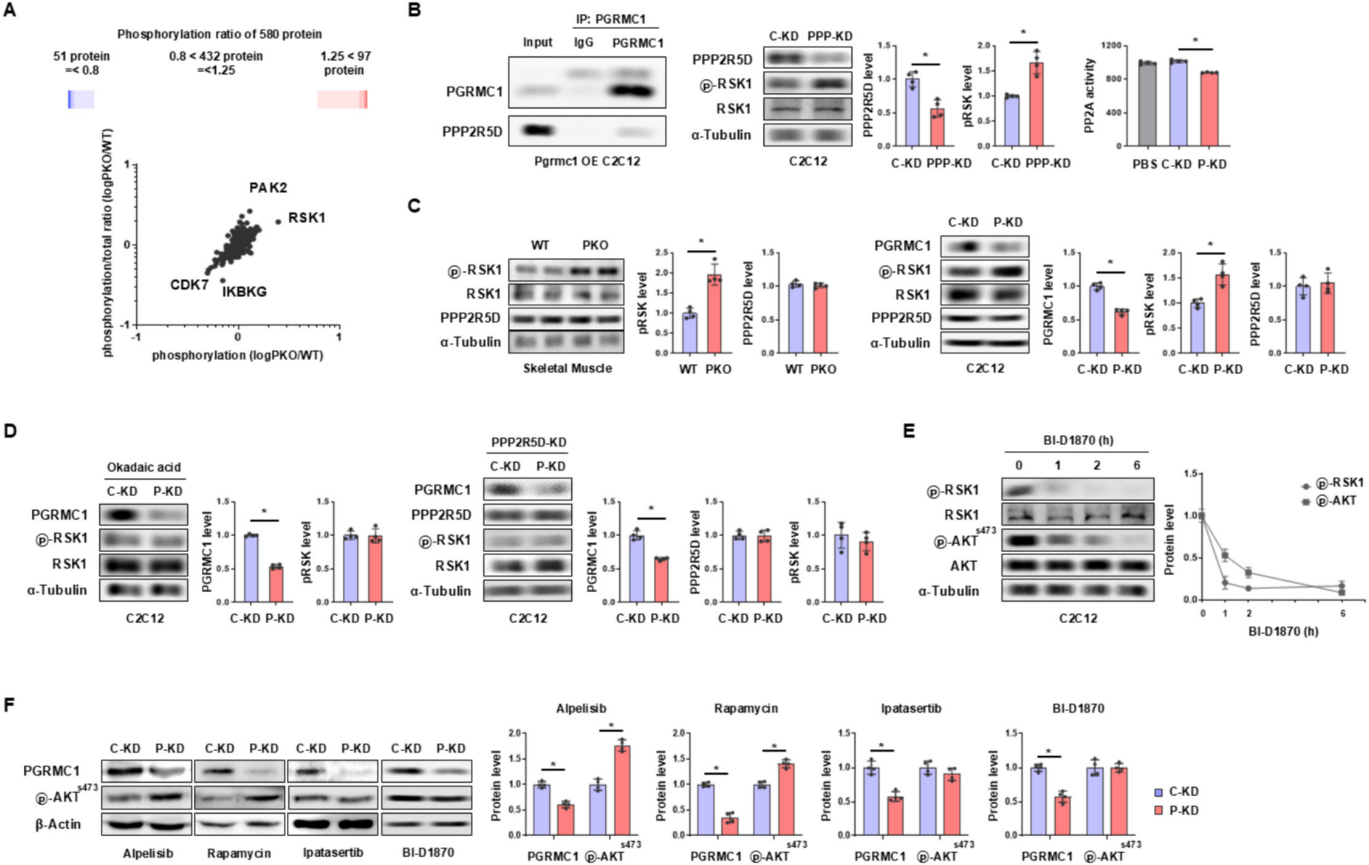
PGRMC1 interacts with PPP2R5D to promote dephosphorylation of RSK1 and inhibit AKT phosphorylation. (A) Phosphoproteome array analysis of WT and PKO skeletal muscles. To enhance experimental efficiency, three samples per group were pooled into one. (B) Co‐immunoprecipitation of PGRMC1 and PPP2R5D. Pgrmc1 was overexpressed in the C2C12 cell line to facilitate efficient co‐immunoprecipitation. Input and IgG were used as positive and negative control, respectively. Western blot analysis and quantification of PPP2R5D, pRSK1 (Ser363) and RSK1 in C‐KD and PPP‐KD C2C12 cells. α‐Tubulin was used as an internal control. PP2A activity (pmoles of phosphate) of PBS, C‐KD and P‐KD C2C12 cells. (C) Western blot analysis and quantification of pRSK1 (ser363), RSK1, and PPP2R5D in WT and PKO skeletal muscles. α‐Tubulin was used as an internal control. Western blot analysis and quantification of PGRMC1, pRSK1 (ser363), RSK1 and PPP2R5D in C‐KD and P‐KD C2C12 cells. α‐Tubulin was used as an internal control. (D) Western blot analysis and quantification of PGRMC1, pRSK1 (ser363) and RSK1 in C‐KD and P‐KD C2C12 cells treated with okadaic acid (10 nM, 96 h). α‐Tubulin was used as an internal control. Western blot analysis and quantification of PGRMC1, PPP2R5D, pRSK1 (ser363) and RSK1 in C‐KD and P‐KD C2C12 cells co‐transfected with Ppp2r5d siRNA (PPP‐KD). α‐Tubulin was used as an internal control. (E) Western blot analysis and quantification of pRSK1 (ser363), RSK1, pAKT (ser473) and AKT in BI‐D1870 treated (0, 1, 2 and 6 h) C2C12 cells. (F) Western blot analysis and quantification of PGRMC1 and pAKT (ser473) in C‐KD and P‐KD A204 cells. β‐Actin was used as an internal control. Cells were treated with alpelisib (1 μM), rapamycin (100 nM), ipatasertib (4 μM), or BI‐D1870 (1 μM) for 96 h. Values represent mean ± SD *p < 0.05, Student's *t*‐test. For cell culture experiments, *n* = 4 independent biological replicates were performed.

To explore the molecular mechanisms by which PGRMC1 promotes development or glucose clearance of skeletal muscle, we conducted a protein array analysis to assess the phosphorylation profile of proteins. Among the 580 proteins analysed, RSK1 exhibited the most dramatic upregulation in PKO skeletal muscle compared to WT skeletal muscle (Figure 2A). Given that RSK1 phosphorylation is regulated by the PP2A enzyme, we further examined the interaction between PPP2R5D, the regulatory subunit of PP2A and PGRMC1 in Pgrmc1‐overexpressing (OE) C2C12 cells, as previous studies have reported an interaction between these two proteins [24]. Co‐immunoprecipitation analysis confirmed a physical interaction between PGRMC1 and PPP2R5D (Figure 2B). Mutations in PPP2R5D have been shown to enhance RSK1 phosphorylation at Ser380, which primes further phosphorylation at Ser363, leading to full activation of RSK1 [25]. Consistently, the knockdown of PPP2R5D (PPP‐KD) in our system resulted in increased phosphorylation of RSK1 (Figure 2B). When *Pgrmc1* was knocked down (P‐KD), PP2A activity was reduced compared to control knockdown (C‐KD) (Figure 2B). Resultantly, RSK1 phosphorylation at Ser363 was significantly elevated in PKO skeletal muscle (Figure 2C), as well as in P‐KD C2C12 cells compared to controls (Figure 2C). Notably, PPP2R5D protein levels remained unchanged between PKO and WT skeletal muscle and between P‐KD and C‐KD cells (Figure 2C), suggesting that PGRMC1 regulates PP2A function without altering PPP2R5D protein levels. Pharmacological inhibition of PP2A with okadaic acid abolished the increase in RSK1 phosphorylation observed in P‐KD cells, as did PPP2R5D knockdown (Figure 2D). Collectively, in the absence of PGRMC1, PP2A activity is compromised, potentially due to altered PPP2R5D conformation, leading to sustained RSK1 phosphorylation.

To explore additional biological mechanisms by which PGRMC1 regulates cellular development and glucose metabolism, we performed Western blot analysis of proteins involved in the mTOR pathway. The mTOR–AKT axis is well established as a key regulator of cellular development and glycolysis [26, 27]. In both normal and T2D skeletal muscle, PKO consistently led to increased AKT phosphorylation at Ser473 (Figure S2A,B). Similarly, P‐KD cells exhibited higher AKT phosphorylation at Ser473 compared to C‐KD cells (Figure S2C‐D). However, mTOR protein levels and phosphorylation status varied across different experimental conditions, suggesting that mTOR may not be the primary mechanism regulated by PGRMC1 (Figure S2). Interestingly, AKT phosphorylation is reported to be associated with RSK1 [28], raising the possibility that PGRMC1‐RSK1 signalling may influence AKT activation in skeletal muscle. To test this, we treated cells with BI‐D1870, an RSK1 inhibitor, in a time‐dependent manner. RSK1 phosphorylation was rapidly suppressed to basal levels within 1 h of treatment, and AKT phosphorylation at Ser473 began to decline at 1 h and reached basal levels by 6 h (Figure 2E). Additionally, we treated C‐KD and P‐KD cells with various inhibitors targeting mTORC1 (alpelisib), mTORC2 (rapamycin), AKT (ipatasertib) and BI‐D1870. Even in the presence of mTORC inhibitors, P‐KD cells continued to exhibit increased AKT phosphorylation compared to C‐KD cells (Figure 2F). However, this increase in AKT phosphorylation was completely abolished upon treatment with the RSK1 inhibitor as well as the AKT inhibitor (Figure 2F). Although there is no direct biochemical evidence that RSK1 phosphorylates AKT, our findings suggest a noncanonical mechanism through which RSK1 contributes to AKT activation independently of PI3K or mTOR pathways.

**FIGURE 3:**
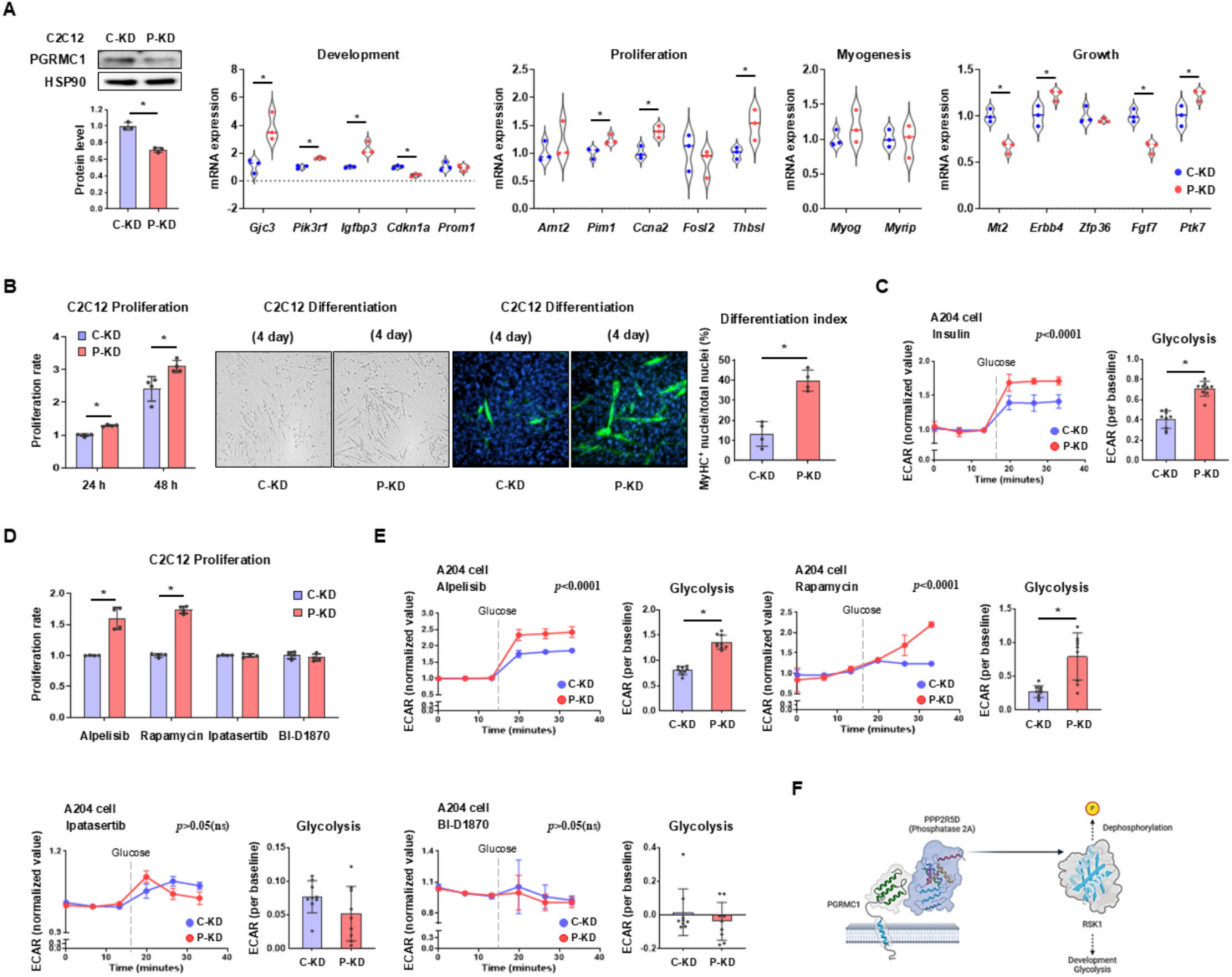
Loss of PGRMC1 promotes skeletal muscle cell proliferation, differentiation, and glycolysis. (A) Western blot analysis and quantification of PGRMC1 in C‐KD and P‐KD C2C12 cells. HSP90 was used as an internal control. mRNA expression levels of enzymes associated with development, proliferation, myogenesis, and growth in C‐KD and P‐KD C2C12 cells. *Rplp0* was used as an internal control. (B) Cell proliferation rates of C‐KD and P‐KD C2C12 cells were measured at 24‐ and 48‐h post‐transfection. Representative images and MyHC immunostainings of differentiated C‐KD and P‐KD C2C12 cells. Differentiation index was calculated by ratio of MyHC positive nuclei per total nuclei. (C) Extracellular acidification rate (ECAR) analysis in C‐KD and P‐KD A204 cells. Values were normalized to baseline. Cells were serum‐starved for 5 h and treated with insulin (100 nM) for 20 min. Glycolysis rate was calculated as the change in ECAR following glucose addition. (D) Proliferation rates of C‐KD and P‐KD C2C12 cells after 96 h of treatment with alpelisib (1 μM), rapamycin (100 nM), ipatasertib (4 μM) or BI‐D1870 (1 μM). (E) Extracellular acidification rate (ECAR) analysis of C‐KD and P‐KD A204 cells. Values were normalized to baseline. Cells were serum‐starved for 5 h and treated with insulin (100 nM) for 20 min. Glycolysis rate was determined based on the ECAR change following glucose treatment. Prior to measurements, cells were treated with alpelisib (1 μM), rapamycin (100 nM), ipatasertib (4 μM) or BI‐D1870 (1 μM) for 96 h. (F) Schematic illustration depicting the regulatory mechanism of PGRMC1 in skeletal muscle development and glycolysis through PPP2R5D and RSK1. Values represent mean ± SD *p < 0.05, Student's *t*‐test. Two‐way ANOVA (column factor) was performed for Seahorse analyses. For cell culture experiments: Panel A, C, E, *n* = 3 independent biological replicates; Panel B, D, *n* = 4 independent biological replicates.

When PGRMC1 protein is knocked down (Figure 3A), developmental or proliferative genes were generally upregulated in P‐KD cells compared to C‐KD cells, while myogenic or growth genes were not increased (Figure 3A). In line with this, P‐KD cells increased proliferation rates and myoblast‐to‐myocyte differentiation rates compared to C‐KD cells (Figure 3B). To minimize the potential interference of cell differentiation in metabolic analysis, we introduced A204 cells, muscle‐derived sarcoma cells with high glycolytic activity [29] but lacking myogenic potential [30]. A Seahorse assay analysis revealed an increased glycolytic rate in P‐KD cells compared to C‐KD cells (Figure 3C). However, when mitochondrial fractions were isolated, ATP production rates between C‐KD and P‐KD mitochondria remained similar, with no significant differences (Figure S3A). Likewise, P‐KD cells did not show increased protein levels of oxidative phosphorylation complexes compared to C‐KD cells (Figure S3B). The Seahorse analysis further confirmed that mitochondrial respiration rates were not elevated in P‐KD cells compared to C‐KD cells (Figure S3C), although fatty acid oxidation rates were lower in P‐KD cells (Figure S3D). Western blot analysis also showed that P‐KD cells did not enhance GLUT4 translocation to the plasma membrane compared to C‐KD cells (Figure S3E). Collectively, these results suggest that while P‐KD cells promote cell differentiation, they do not broadly activate cellular metabolism, except for glycolysis, when differentiation is restricted.

Although cell differentiation could not be assessed due to the strong inhibitory effects of these compounds, cell proliferation induced by PGRMC1 loss was abolished by both the AKT and RSK1 inhibitors, while mTORC inhibitors had no effect on this trend (Figure 3D). Furthermore, PGRMC1 loss‐induced glycolysis was also suppressed by both AKT and RSK1 inhibitors, while mTORC inhibitors had no significant effect (Figure 3E). Together, PGRMC1 regulates AKT phosphorylation through RSK1, establishing a causal relationship in the regulation of cellular development and glycolysis (Figure 3F).

### 3.3 11α‐OHP, a PGRMC1 Lowering Drug, Improves Glucose Homeostasis and Enhances Insulin Sensitivity

**FIGURE 4:**
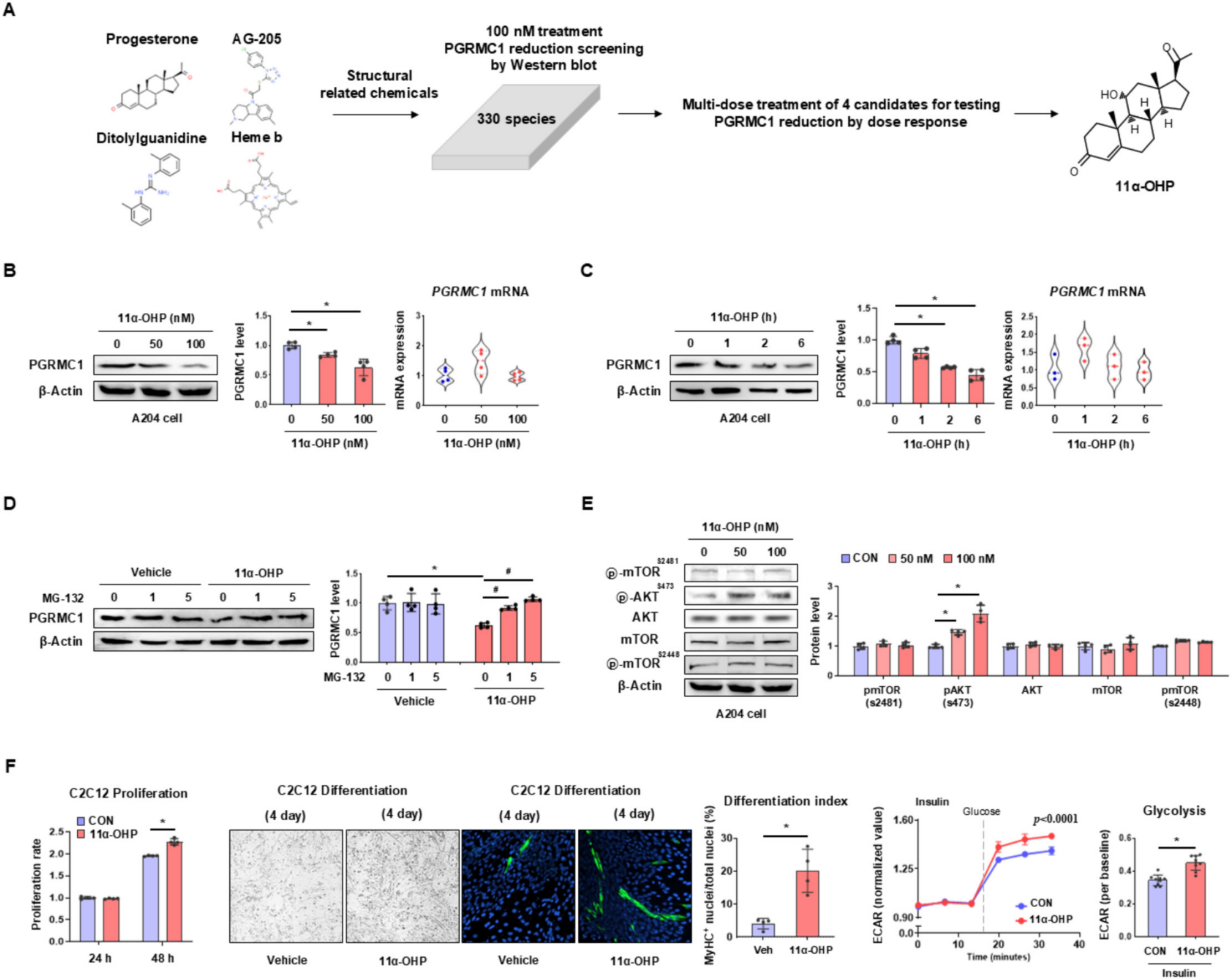
Pharmacological inhibition of PGRMC1 enhances skeletal muscle cell proliferation, differentiation, and glycolysis. (A) Schematic diagram illustrating the discovery process of the Pgrmc1‐lowering drug. In vitro hit screening and dose–response treatments were performed. (B, C) Western blot analysis and quantification of PGRMC1 in A204 cells treated by 11α‐OHP at various doses (for 24 h) or in a time‐dependent manner (at 100 nM for the indicated time points). β‐Actin was used as an internal control. mRNA expression levels of *PGRMC1* in A204 cells treated by 11α‐OHP. *RPLP0* used as an internal control. (D) Western blot analysis and quantification of PGRMC1 in A204 cells treated with MG‐132 (a proteasomal degradation inhibitor). β‐Actin was used as an internal control. (E) Western blot analysis and quantification of mTOR and AKT‐related proteins in A204 cells. β‐Actin was used as an internal control. (F) Cell proliferation rates of C2C12 cells treated with vehicle or 11α‐OHP after 24 and 48 h of treatment. Representative images and MyHC immunostainings of differentiated C2C12 cells treated with vehicle or 11α‐OHP. Differentiation index was calculated by ratio of MyHC positive nuclei per total nuclei. Extracellular acidification rate (ECAR) analysis of A204 cells treated with vehicle or 11α‐OHP. Values were normalized to baseline. Cells were serum‐starved for 5 h and treated with insulin (100 nM) for 20 min. Glycolysis rate was calculated based on ECAR changes upon glucose treatment. Values represent mean ± SD *p < 0.05, Student's *t*‐test or one‐way ANOVA followed by Tukey's multiple comparisons test. Two‐way ANOVA (column factor) was performed for Seahorse analyses. For cell culture experiments: Panels B–F (without seahorse), *n* = 4 independent biological replicates; Panel F (seahorse), *n* = 3 independent biological replicates.

We screened 330 species of chemicals structurally related to known Pgrmc1 or sigma‐2 receptor modulators, including progesterone [17], AG‐205 [31], ditolylguanidine [32] and heme b [33]. To identify chemicals that act on skeletal muscle PGRMC1, we used the A204 cell line for screening. From an initial screening of PGRMC1 protein levels after treatment with 100 nM of these chemicals, we identified four potential candidates as Pgrmc1‐lowering compounds. Following a dose–response analysis of these four chemicals, we determined that 11α‐OHP was the most effective PGRMC1 suppressor (Figure 4A). Western blot analysis confirmed that 11α‐OHP decreased PGRMC1 protein levels in a dose‐dependent manner (Figure 4B). Additionally, it reduced PGRMC1 protein levels in a time‐dependent manner, showing a rapid effect within 2 to 6 h (Figure 4C). Notably, PGRMC1 mRNA levels remained unchanged, suggesting that the reduction in PGRMC1 protein was not due to transcriptional regulation (Figure 4B,C). Instead, the decrease in PGRMC1 protein levels was associated with proteasomal degradation, as treatment with MG‐132, a proteasomal degradation inhibitor, restored PGRMC1 levels in 11α‐OHP‐treated cells (Figure 4D). Furthermore, 11α‐OHP increased AKT phosphorylation in a dose‐dependent manner, like the P‐KD condition (Figure 4E). In functional assays, 11α‐OHP promoted myoblast proliferation and differentiation in C2C12 cells and enhanced glycolytic activity in A204 cells likethe P‐KD condition (Figure 4F).

**FIGURE 5:**
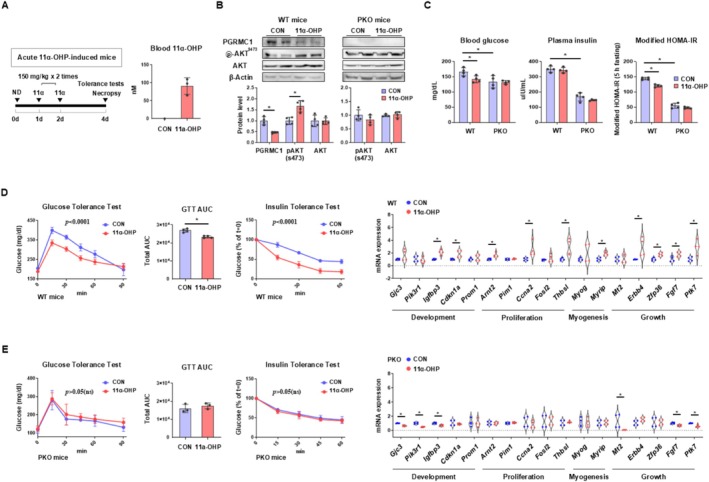
In vivo treatment with a PGRMC1 inhibitor enhances glucose clearance and insulin responsiveness. (A) Experimental timeline for acute 11α‐OHP treatment (150 mg/kg, administered twice). Blood concentration of 11α‐OHP at necropsy. (B) Western blot analysis and quantification of PGRMC1, pAKT, and AKT in skeletal muscles of WT or PKO mice treated with either vehicle or 11α‐OHP. β‐Actin was used as an internal control. (C) Blood glucose (mg/dL), plasma insulin (uIU/mL) and modified HOMA‐IR (blood glucose × plasma insulin/405) levels of WT and PKO mice treated with vehicle or 11α‐OHP. (D, E) Glucose tolerance test (GTT) and insulin tolerance test (ITT) in WT and PKO mice treated with vehicle or 11α‐OHP. The area under curve (AUC) for GTT was calculated. mRNA expression levels of genes related to development, proliferation, myogenesis and growth in skeletal muscles of WT or PKO mice treated with vehicle or 11α‐OHP. *Rplp0* was used as an internal control. Mice used for experiments: 4 for each group. Values represent mean ± SD *p < 0.05, Student's *t*‐test or one‐way ANOVA followed by Tukey's multiple comparisons test. Two‐way ANOVA (column factor) was performed for GTT and ITT.

To determine whether 11α‐OHP exhibits biologically substantial effects in vivo, we administered 150 mg/kg of the compound twice over two consecutive days in mice (Figure 5A). LC/MS analysis confirmed that the plasma concentration of 11α‐OHP at the time of necropsy was approximately 90 nM, which closely matched the concentration used in our in vitro experiments (Figure 5A). Notably, endogenous 11α‐OHP was undetectable in control mice (*n* = 1) (Figure 5A). Western blot analysis confirmed that PGRMC1 levels decreased and pAKT levels increased in wild‐type mice following 11α‐OHP treatment. However, in PKO mice, where PGRMC1 protein is absent, 11α‐OHP did not increase AKT phosphorylation (Figure 5B). Metabolic analysis revealed that 11α‐OHP lowered blood glucose levels without affecting plasma insulin levels in WT mice. In contrast, PKO mice showed no changes in blood glucose or insulin levels upon 11α‐OHP treatment (Figure 5C). Consistently, modified HOMA‐IR levels decreased in WT mice but remained unchanged in PKO mice after 11α‐OHP treatment (Figure 5C). Western blot analysis revealed a consistent increase of glycolytic enzymes following 11α‐OHP treatment, but only in WT mice, not PKO mice (Figure S4). Furthermore, in GTT and ITT, WT mice treated with 11α‐OHP exhibited significant reductions in blood glucose levels, whereas PKO mice did not show any improvements (Figure 5D,E). Gene expression analysis of skeletal muscle tissues from WT mice treated with 11α‐OHP revealed upregulation of genes related to development, proliferation, myogenesis and muscle growth (Figure 5D). However, PKO mice treated with 11α‐OHP did not induce any significant increases in gene expression (Figure 5E). Taken together, these findings demonstrate that 11α‐OHP enhances glucose homeostasis and promotes skeletal muscle development in vivo by lowering PGRMC1 levels. Additionally, the lack of response in PKO mice indicates that 11α‐OHP has minimal off‐target effects, further supporting its specificity as a PGRMC1‐lowering agent.

### 3.4 11α‐OHP Suppresses T2D Progression and Promotes Recovery to a Normal State

**FIGURE 6:**
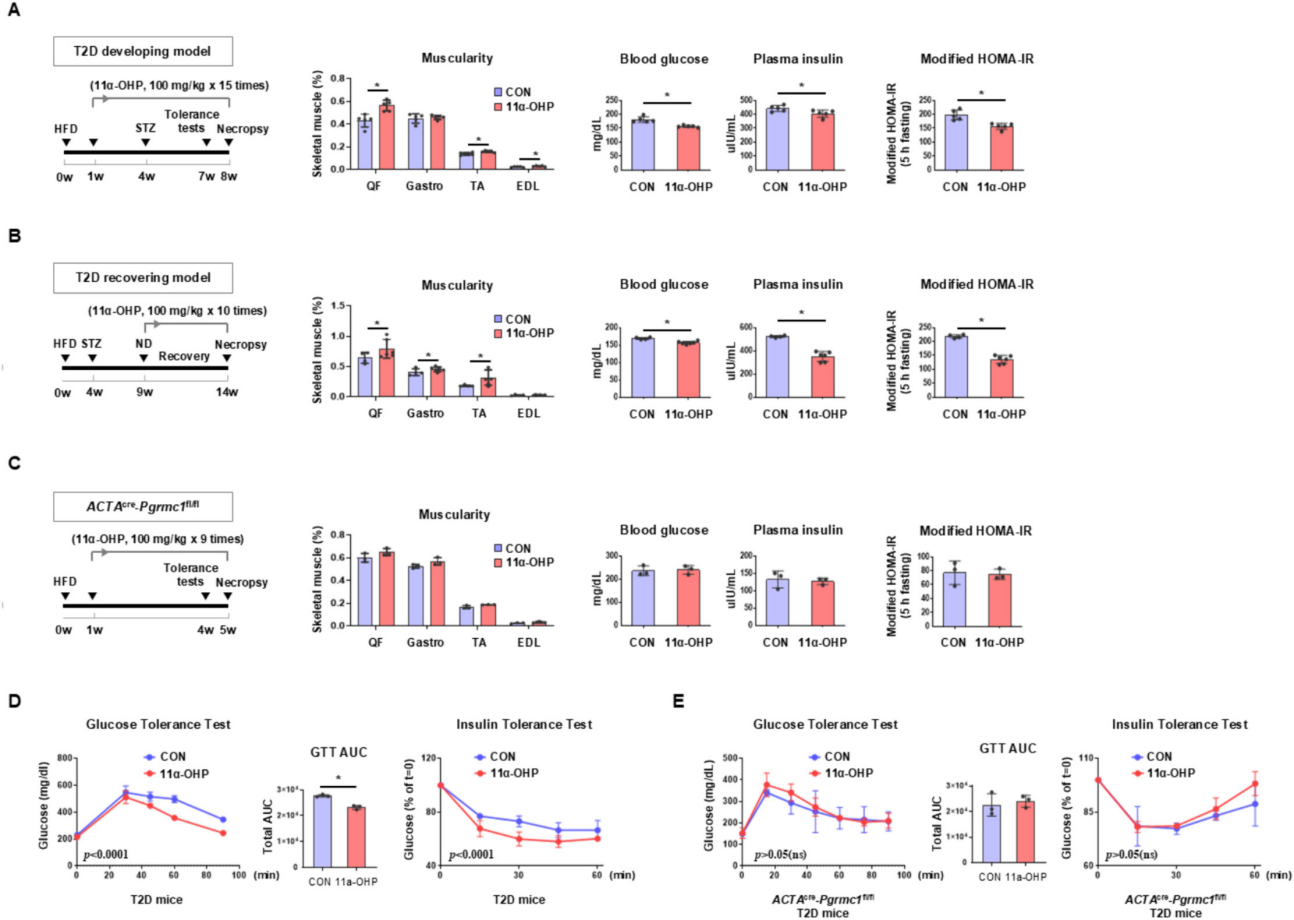
Pharmacological PGRMC1 inhibition improves metabolic outcomes in HFD‐STZ mouse models. (A, B) Experimental timeline for 11α‐OHP treatment (100 mg/kg) in mice developing or recovering from T2D. Individual skeletal muscle weights in the hindlimb (quadriceps femoris, QF; gastrocnemius, Gastro; tibialis anterior, TA; extensor digitorum longus, EDL) normalized to BW (muscularity, %) in T2D‐developing or recovering mice treated with vehicle or 11α‐OHP. Blood glucose (mg/dL) and plasma insulin (μIU/mL) levels in fasted (5 h) T2D‐developing or recovering mice. Modified HOMA‐IR values (blood glucose × plasma insulin/405) indicating insulin resistance in T2D‐developing or recovering mice treated with vehicle or 11α‐OHP under fasting conditions (5 h, modified to simulate a resting state). (C) Experimental schedule for inducing metabolic dysregulation in *ACTA* ^cre^‐*Pgrmc1* ^fl/fl^ mice. Skeletal muscle weight relative to body weight in *ACTA* ^cre^‐*Pgrmc1* ^fl/fl^ mice treated with either vehicle or 11α‐OHP. Skeletal muscle weight relative to body weight of *ACTA* ^cre^‐*Pgrmc1* ^fl/fl^ mice treated with vehicle or 11α‐OHP. Blood glucose (mg/dL) and plasma insulin (μIU/mL) levels in *ACTA* ^cre^‐*Pgrmc1* ^fl/fl^ mice treated with either vehicle or 11α‐OHP. (D) Glucose tolerance test (GTT) and insulin tolerance test (ITT) in mice treated with vehicle or 11α‐OHP. The area under curve (AUC) for GTT was calculated. (E) Glucose tolerance test (GTT) and insulin tolerance test (ITT) in *ACTA* ^cre^‐*Pgrmc1* ^fl/fl^ mice treated with either vehicle or 11α‐OHP. Mice used in the experiment: 5 for T2D‐developing control, 5 for T2D‐developing 11α‐OHP, 4 for T2D‐recovering control, 6 for T2D‐recovering 11α‐OHP, 3 for each *ACTA* ^cre^‐*Pgrmc1* ^fl/fl^ group. Values represent mean ± SD *p < 0.05, Student's *t*‐test or one‐way ANOVA followed by Tukey's multiple comparisons test. Two‐way ANOVA (column factor) was performed for GTT and ITT.

To investigate whether in vivo administration of 11α‐OHP exerts antidiabetic effects in disease models, we established three experimental models for assessments: 11α‐OHP administration during (1) development and (2) the recovery phase in the HFD‐STZ model, as well as (3) the development of T2D in the *db/db* model. First, 11α‐OHP was administered during T2D development, as illustrated in Figure 6A. The muscularity was significantly increased in 11α‐OHP‐treated mice (Figure 6A). For the recovery phase, mice first underwent T2D development and were subsequently rehabilitated through normal diet feeding. During the recovery period, 11α‐OHP was administered (Figure 6B). Like the T2D developing model, 11α‐OHP treatment significantly increased muscularity (Figure 6B). In both models, 11α‐OHP‐treated mice exhibited lower blood glucose and plasma insulin levels compared to vehicle‐treated mice. Consistently, the modified HOMA‐IR levels in 11α‐OHP‐treated mice were significantly reduced (Figure 6A,B). To evaluate whether 11α‐OHP enhances insulin sensitivity by improving skeletal muscle metabolism, we performed several analyses. LC/MS analysis revealed that glucose and its metabolites were overall increased in the skeletal muscle of 11α‐OHP‐treated mice during T2D development (Figure S5A), suggesting that blood glucose clearance by skeletal muscle was enhanced. In both the T2D development and recovery phases, SDH enzyme activity was significantly elevated in skeletal muscles of 11α‐OHP‐treated mice (Figure S5B,C), indicating increased mitochondrial metabolism. Furthermore, immunostaining results showed a significant increase in type IA fibre abundance in skeletal muscle during both T2D development and recovery (Figure S5D‐E). Given the observed increase in muscularity and the metabolic activation induced by 11α‐OHP, we investigated whether skeletal muscle PGRMC1 is essential for mediating the effects of 11α‐OHP on glucose homeostasis and insulin sensitivity. To explore this, *ACTA* ^cre^‐*Pgrmc1* ^fl/fl^ mice were administered 11α‐OHP, as illustrated in Figure 6C. 11α‐OHP treatment on *ACTA* ^cre^‐*Pgrmc1* ^fl/fl^ mice did not exhibit increases in muscularity (Figure 6C). Furthermore, 11α‐OHP treatment on *ACTA* ^cre^‐*Pgrmc1* ^fl/fl^ mice did not alter any blood glucose or plasma insulin levels (Figure 6C). To further validate the effects of 11α‐OHP on glucose homeostasis and insulin sensitivity, we conducted GTT and ITT. During HFD‐STZ progression, 11α‐OHP‐treated mice exhibited significantly lower blood glucose levels compared to controls in both GTT and ITT (Figure 6D). However, *ACTA* ^cre^‐*Pgrmc1* ^fl/fl^ mice treated with 11α‐OHP did not exhibit any improvements in GTT or ITT compared to vehicle‐treated controls (Figure 6E). Taken together, these results indicate that 11α‐OHP regulates whole‐body glucose homeostasis and insulin sensitivity primarily through modulation of skeletal PGRMC1 protein.

**FIGURE 7:**
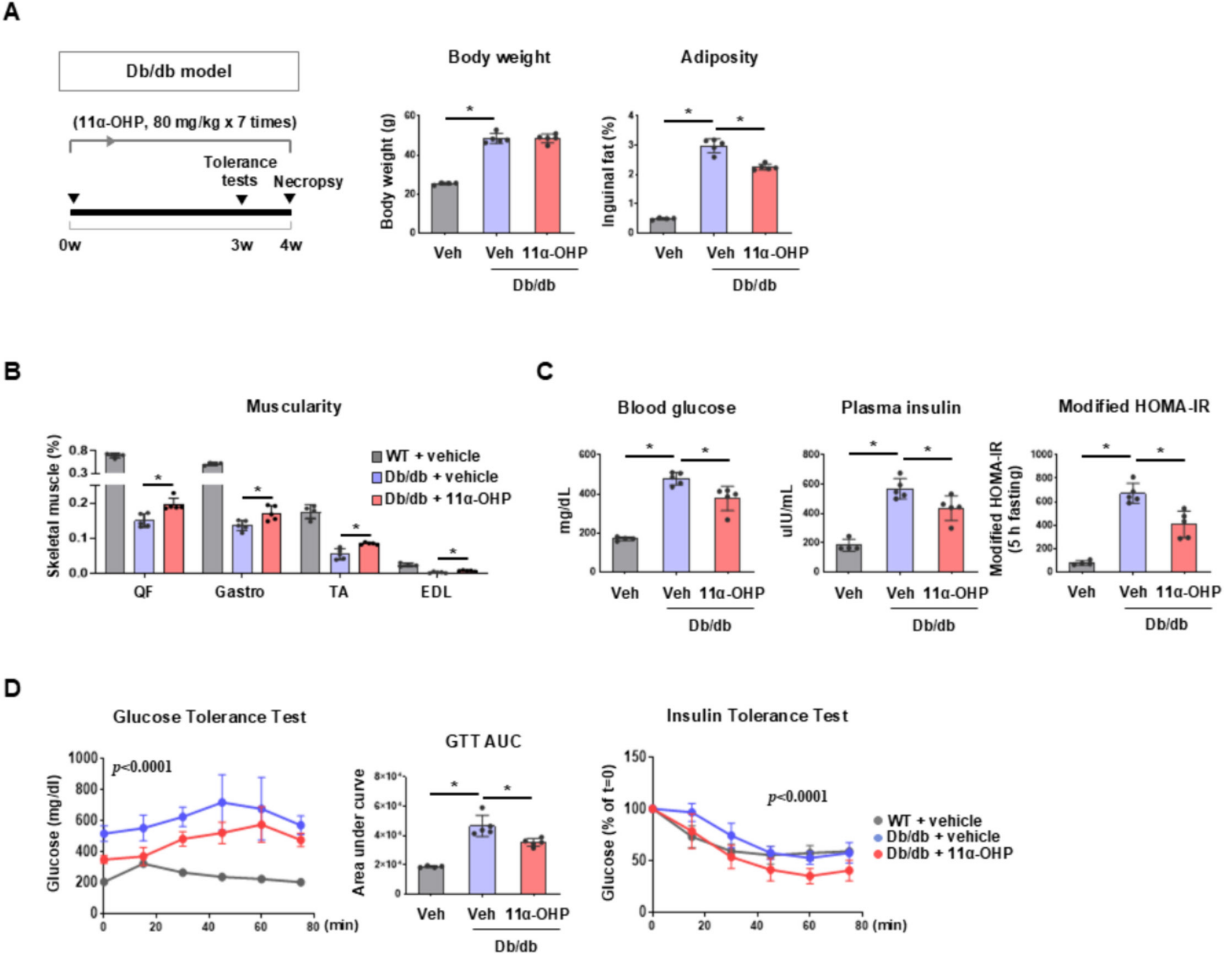
Pharmacological inhibition of PGRMC1 improves skeletal muscle mass and insulin sensitivity in db/db mice. (A) Body weight (BW, g) and inguinal adipose tissue weight per BW (adiposity, %) of *db/db* mice treated with vehicle or 11α‐OHP. (B) Individual skeletal muscle weights in the hindlimb (quadriceps femoris, QF; gastrocnemius, Gastro; tibialis anterior, TA; extensor digitorum longus, EDL) normalized to BW (muscularity, %) of *db/db* mice treated with vehicle or 11α‐OHP. (C) Blood glucose (mg/dL) and plasma insulin (uIU/mL) levels of *db/db* mice under fasted conditions (5 h). Modified HOMA‐IR values (blood glucose x plasma insulin/405) representing insulin resistance of *db/db* mice treated with vehicle or 11α‐OHP under fasting conditions (5 h, modified for resting state simulation). (D) Glucose tolerance test (GTT) and insulin tolerance test (ITT) in *db/db* mice treated with vehicle or 11α‐OHP. The area under curve (AUC) for GTT was calculated. Mice used for the experiment: 4 for control, 5 for *db/db* control and 5 for *db/db* 11α‐OHP. Values represent mean ± SD *p < 0.05, Student's *t*‐test or one‐way ANOVA followed by Tukey's multiple comparisons test. Two‐way ANOVA (column factor) was performed for GTT and ITT.

To assess the therapeutic potential of 11α‐OHP in an advanced metabolic disease model, we utilized *db/db* mice and administered 11α‐OHP (Figure 7A). Treatment began at 6 weeks of age and continued for 18 days. Food intake was unaffected by 11α‐OHP (Figure S6A), but blood glucose levels were significantly reduced starting on day 14 (Figure S6B). At the end of the study, relative skeletal muscle mass was increased in the 11α‐OHP treated group (Figure 7B). In contrast, adiposity was decreased in the 11α‐OHP treated group and the body weight was unchanged (Figure 7A). 11α‐OHP treated mice displayed significantly lower blood glucose and plasma insulin levels, along with decreased modified HOMA‐IR scores (Figure 7C). Glucose tolerance and insulin sensitivity were also improved in 11α‐OHP treated mice, as evidenced by GTT and ITT results (Figure 7D). The efficacy of 11α‐OHP in both HFD‐STZ and *db/db* models, despite their distinct pathophysiological mechanisms, suggests its broad potential to restore metabolic homeostasis in diverse diabetic contexts. To examine possible off‐target effects of systemic Pgrmc1 inhibition, we analysed skeletal muscle in whole‐body PKO mice [19] under HFD‐STZ induced T2D. These mice exhibited increased skeletal muscle development, higher abundance of type I fibres and enhanced activation of glycolytic and oxidative phosphorylation enzymes, including SDH and GLUT4 translocation. Transcriptomic analyses further supported the upregulation of genes associated with skeletal muscle development (Figure S7). Collectively, our results indicate that systemic PGRMC1 inhibition by 11α‐OHP improves metabolic outcomes even in advanced stages of diabetes, without apparent toxicity or non‐muscle‐specific adverse effects, underscoring its therapeutic potential.

## 4 Discussion

While lifestyle modification remains the cornerstone of T2D management, pharmacological interventions have significantly advanced over the past decades. Most recently, pharmaceutical companies have developed anti‐diabetic treatment through anti‐obesity approaches like GLP‐1R agonists [34] or co‐agonists for glucose‐dependent insulinotropic polypeptide receptor (GIPR) and GLP‐1R [35]. However, the rapid rise of anti‐obesity strategies has raised concerns about unintended skeletal muscle loss [35]. Given that skeletal muscle is a primary site for glucose disposal and insulin action, pharmaceutical companies have begun exploring therapies that target muscle metabolism directly [36]. In this context, we propose skeletal PGRMC1 as a novel muscle‐specific therapeutic target for T2D. By promoting RSK‐AKT‐mediated glycolysis and myogenesis, PGRMC1 inhibition improves glucose homeostasis and muscle function without systemic adverse effects.

Given its diverse cellular functions, including roles as a cargo protein [37] and as a chaperone [38], we hypothesized that PGRMC1 functions as a protein–protein interaction hub that regulates intracellular signalling relevant to glucose metabolism. A phospho‐protein array revealed a marked increase in RSK1 phosphorylation in Pgrmc1‐deficient skeletal muscle. Consistently, RNA‐seq analysis showed that genes involved in developmental processes were significantly upregulated in PKO compared with WT muscle. Western blot analysis further demonstrated that AKT phosphorylation was consistently elevated in Pgrmc1‐deficient cells, whereas mTOR phosphorylation remained unchanged. Building on previous evidence that RSK can phosphorylate AKT [28], our experiments identified a PP2A–RSK1–AKT axis under the control of PGRMC1. A previous study reported that PGRMC1 interacts with multiple proteins, including PPP2R2B and PPP2R5D [24], both of which are regulatory subunits of PP2A, a phosphatase complex involved in the dephosphorylation of various proteins [39]. Of these, PPP2R5D has been specifically linked to RSK1 phosphorylation [25]. Our co‐immunoprecipitation experiments reconfirmed a direct physical interaction between PGRMC1 and PPP2R5D. When PP2A activity was inhibited, the increase in RSK1 phosphorylation in P‐KD cells was abolished. Upon treatment with the RSK1 inhibitor BI‐D1870, RSK1 phosphorylation decreased first, followed by a delayed reduction in AKT phosphorylation, supporting a sequential RSK1–AKT signalling relationship. Additionally, the inhibition of RSK1 abrogated the increase in AKT phosphorylation, cell proliferation and glycolysis in P‐KD cells. Collectively, these findings uncover a previously unrecognized mechanism whereby PGRMC1 regulates skeletal muscle development and glycolytic metabolism through the PP2A–RSK1–AKT axis.

In comparison with our earlier work employing whole‐body PKO mice, several points merit further discussion. In our previous study, we demonstrated that Pgrmc1 loss in HFD‐fed mice exacerbated hepatic steatosis and steatohepatitis through enhanced de novo lipogenesis, a liver‐specific metabolic pathway that generates malonyl‐CoA for fatty acid synthesis. This phenotype was accelerated by increased levels of mature SREBP‐1 protein, a transcriptional activator of de novo lipogenesis, which may result from structural instability of the putative PGRMC1/INSIG‐1/SCAP/SREBP‐1 complex in the endoplasmic reticulum of PKO livers [19]. Although fatty livers are typically associated with insulin resistance [40], the metabolic benefit conferred by skeletal Pgrmc1 loss outweighed the potential disadvantage from hepatic Pgrmc1 loss, thereby ameliorating systemic insulin resistance in T2D PKO mice (Figure S8). In addition, we reported that Pgrmc1 loss in HFD‐fed mice promoted cardiac lipotoxicity [18]. Specifically, Pgrmc1 deficiency in cardiac muscle cells reduced fatty acid oxidation and mitochondrial respiration, whereas Pgrmc1 loss in skeletal muscle cells did not significantly alter mitochondrial respiration rates, despite a decrease in fatty acid oxidation. This discrepancy may stem from the different metabolic dependencies of the heart and skeletal muscle on mitochondrial respiration for ATP production. The heart derives approximately 70%–90% of its ATP from fatty acid oxidation, with only 10%–30% from glucose and lactate oxidation, indicating that glycolysis plays a minimal role in cardiac ATP generation [41]. In contrast, skeletal muscle can rapidly increase ATP demand during intense contraction, often surpassing the capacity of mitochondrial respiration. Under such conditions, energy production initially relies on the phosphocreatine system, which provides immediate ATP buffering, and subsequently shifts toward glycolysis, enabling a faster rate of ATP regeneration [42]. This metabolic prioritization allows skeletal muscle to meet acute energetic demands through non‐mitochondrial pathways such as glycolysis in PKO mice. These distinct properties suggest that skeletal muscle is less susceptible to the deleterious effects of Pgrmc1 loss observed in other tissues, thereby providing a rationale for targeting skeletal muscle Pgrmc1 as a therapeutic strategy.

Regular physical activity increases muscle mass and prevents the onset of T2D [43]. In addition, healthy skeletal muscle improves insulin sensitivity owing to the abundance of oxidative fibres [44]. In line with these concepts, we anticipate that 11α‐OHP may provide clinical benefits by improving skeletal muscle health, as it not only promotes recovery of muscle mass but also enhances oxidative fibre abundance and oxidative capacity during both the development and recovery phases of T2D. Given its distinct mechanism of action, 11α‐OHP may act synergistically with GLP‐1 receptor agonists. Notably, intramuscular administration of 11α‐OHP improved glucose clearance and insulin sensitivity (Figure S9), suggesting that 11α‐OHP could be used with convenience comparable to insulin. Despite these promising results, it should be noted that 11α‐OHP inhibits PGRMC1 across multiple organs, and thus, potential systemic adverse effects cannot be fully excluded. However, no evidence of hepatotoxicity or nephrotoxicity was observed, as plasma ALT and BUN levels remained unchanged following 11α‐OHP treatment (Figure S10). In addition, whole‐body ablation of Pgrmc1 still enhanced skeletal muscle development and metabolic activation, indicating that muscle PGRMC1 plays a predominant role. Nevertheless, more targeted strategies to selectively inhibit PGRMC1 in skeletal muscle are needed. As shown in Figure S11, where AAV6 was employed to enhance muscle specificity, gene therapy approaches incorporating tissue‐selective modulation of PGRMC1 should be further explored.

In conclusion, our study identifies PGRMC1 as a key regulator of skeletal muscle development and glucose metabolism and highlights its inhibition as a potential therapeutic strategy for T2D. Targeting PGRMC1 may confer muscle‐specific metabolic benefits, with possible implications beyond T2D to other muscle‐related metabolic disorders. Future work should focus on optimizing muscle‐selective delivery approaches and further evaluating efficacy and safety in clinical settings.

## Conflicts of Interest

The authors declare no conflicts of interest.

## Supporting information


**Figure S1:** Generation of Pgrmc1^fl/fl^ mice. sgRNAs targeting Pgrmc1 introns were used to insert loxP sequences, generating the Pgrmc1 floxed allele.
**Figure S2:** Increased AKT phosphorylation in PKO skeletal muscle and P‐KD cells. (A‐B) Western blot analysis and quantification of pmTOR (ser2481), mTOR, phosphorylated mTOR (ser2448), pAKT (ser473) and AKT in WT and PKO skeletal muscles under normal and type 2 diabetes (T2D) conditions. Mice used for the experiment: 4 for normal WT, 4 for normal PKO, 6 for T2D WT and 6 for T2D PKO. (C‐D) Western blot analysis and quantification of PGRMC1, pmTOR (ser2481), mTOR, phosphorylated mTOR (ser2448), pAKT (ser473) and AKT in C‐KD and P‐KD C2C12 or A204 cells. Values represent means ± SD *, *p* < 0.05. Student's *t*‐test was performed. For cell culture experiments, *n* = 4 independent biological replicates were performed.
**Figure S3:** Metabolic phenotype of P‐KD cells. (A) Relative ATP levels in isolated mitochondria from C‐KD and P‐KD A204 cells. ADP (10 μM) was incubated for 1 h at 37°C. (B) Western blot analysis of PGRMC1 and oxidative phosphorylation protein complexes (NDUFB8, CI; SDH8, CII; UQCRC2, CIII; MTCO1, CIV; ATP5A, CV) in C‐KD and P‐KD A204 cells. β‐Actin was used as an internal control. (C) Oxygen consumption rates (OCR) in C‐KD and P‐KD A204 cells were measured using a flux analyser with chemical treatments (oligomycin [Omy]; carbonyl cyanide‐p‐trifluoromethoxyphenylhydrazone [FCCP]; rotenone [Rot]; antimycin [Ant]). Values were normalized to baseline. ATP production rate (OCR change in response to Omy) and maximal respiration rate (OCR change in response to Rot/Ant) were calculated. (D) OCR in C‐KD and P‐KD A204 cells were measured using a flux analyser under the same chemical treatments in media containing BSA‐conjugated palmitate (200 μM). Values were normalized to baseline. ATP production rate (OCR change in response to Omy) and maximal respiration rate (OCR change in response to Rot/Ant) were calculated. Cells were starved for 5 h and treated with insulin (100 nM) for 30 min. (E‐F) Western blot analysis and quantification of GLUT4 in A204 cells. Cells were either untreated or treated with insulin (100 nM). α‐Tubulin or NKA was used as an internal control. Values represent mean ± SD. *p < 0.05, Student's t‐test and two‐way ANOVA (column factor) test were performed. For cell culture experiments: Panel A, *n* = 4 independent biological replicates; Panel B‐F, *n* = 3 independent biological replicates.
**Figure S4:** Increased glycolytic enzyme and glucose uptake protein levels in skeletal muscles of 11α‐OHP‐treated mice only in the presence of PGRMC1. Western blot analysis and quantification of glycolytic enzymes (HK2, PKM1/2 and PDH) and the glucose transporter GLUT4 in skeletal muscles of WT and PKO mice treated with 11α‐OHP. Mice used for experiments: 4 for each group. HSP90 and NKA were used as internal controls. Values represent means ± SD. *, *p* < 0.05. Student's *t*‐test was performed.
**Figure S5:** Increased glycolytic metabolites, mitochondrial metabolism and oxidative fibre abundance in 11α‐OHP‐treated skeletal muscle. (A) Quantification of glucose and glucose metabolites in WT and PKO skeletal muscles using LC/MS. The unit represents the relative ratio, calculated as the analyte peak area divided by the internal standard peak area and normalized to tissue weight (mg). (B, C) SDH staining of skeletal muscle from vehicle‐treated and 11α‐OHP‐treated mice in both the developing and recovery stages of T2D. (D, E) Immunostaining of type IA, IIA and IIB muscle fibres in skeletal muscle from vehicle‐treated and 11α‐OHP‐treated mice in both the developing and recovery stages of T2D. Mice used in the experiment: 5 for T2D‐developing control, 5 for T2D‐developing 11α‐OHP, 4 for T2D‐recovering control and 6 for T2D‐recovering 11α‐OHP. Values represent means ± SD. *, *p* < 0.05. Student's *t*‐test was performed.
**Figure S6:** Monitoring of diet intake and blood glucose levels of Db/db mice. (A) Average diet intake (g) per group in Db/db mice. Diet intake was measured collectively for each group, not individually. (B) Blood glucose level monitoring throughout the treatment period. Values represent means ± SD. *, *p* < 0.05. Mice used for the experiment: 4 for control, 5 for *db/db* control and 5 for *db/db* 11α‐OHP. One‐way ANOVA followed by Tukey's multiple comparisons test were performed.
**Figure S7:** Systemic PKO retains metabolic activation of skeletal muscle. (A) Experimental schedule for type 2 diabetes (T2D) induction and representative dual X‐ray absorptiometry (DEXA) images of T2D WT and PKO mice. Streptozotocin (STZ, 30 mg/kg) was administered intraperitoneally. DEXA measurements showing lean mass relative to body mass (%) and fat mass (%) in WT and PKO mice. Mice used for the analysis: 4 per group. (B) Immunostaining of type IA, IIA and IIB muscle fibres in WT and PKO skeletal muscles. (C) Western blot analysis and quantification of key enzymes involved in glycolysis and glucose uptake in WT and PKO skeletal muscles. HSP90 and membrane NKA were used as internal controls. Western blot analysis and quantification of key enzymes involved in oxidative phosphorylation in WT and PKO skeletal muscles. HSP90 was used as internal control. (D) SDH staining of the tibialis anterior (TA) skeletal muscle in WT and PKO mice. SDH enzymes are visualized in purple. Mice used for the experiment: 6 per group. (E) Immunostaining of GLUT4 (red) in WT and PKO skeletal muscles. DAPI (blue) was used as a nuclear counterstain. (F) Top 10 biological processes showing significant differences in PKO skeletal muscle based on RNA sequencing analysis. Heatmap of differentially expressed mRNAs related to development, proliferation, myogenesis and growth in WT and PKO skeletal muscle from RNA sequencing analysis. Fold changes are indicated by colour (red, increase; blue, decrease). Mice used for RNA sequencing analysis: 3 per group. Values represent means ± SD. *, *p* < 0.05. Student's *t*‐test was performed.
**Figure S8:** Systemic PKO improves glucose clearance and insulin responsiveness in T2D. Experimental schedule for type 2 diabetes (T2D) induction. Streptozotocin (STZ, 30 mg/kg) was administered intraperitoneally. Glucose tolerance test (GTT) and insulin tolerance test (ITT) in WT and PKO mice. Mice used in experiments: 4 (WT) and 3 (PKO). Values represent mean ± SD. *p < 0.05, Student's *t*‐test. Two‐way ANOVA (column factor) was performed for GTT and ITT.
**Figure S9:** Enhanced glucose clearance and insulin sensitivity by intramuscular injection of 11α‐OHP. (A) Schematic representation of the intramuscular injection of 11α‐OHP. Gross image of sacrificed mice displaying Cy5‐labelled 11α‐OHP as a blue tint. In vivo imaging showing the distribution of Cy5‐labelled 11α‐OHP. (B) Blood glucose levels, plasma insulin levels and modified HOMA‐IR in vehicle‐treated and 11α‐OHP‐treated mice. (C) Glucose tolerance test (GTT) and insulin tolerance test (ITT) in vehicle‐treated and 11α‐OHP‐treated mice. Mice used in experiments: 4 per group. Values represent mean ± SD. *p < 0.05, Student's *t*‐test. Two‐way ANOVA (column factor) was performed for GTT and ITT.
**Figure S10:** Blood markers of hepatotoxicity and nephrotoxicity in 11α‐OHP treated mice. (A) Plasma ALT levels of vehicle‐treated and 11α‐OHP‐treated mice in both the developing and recovery stages of T2D. (B) Plasma BUN levels of vehicle‐treated and 11α‐OHP‐treated mice in both the developing and recovery stages of T2D. Mice used in the experiment: 5 for T2D‐developing control, 5 for T2D‐developing 11α‐OHP, 4 for T2D‐recovering control and 6 for T2D‐recovering 11α‐OHP. Values represent means ± SD. *, *p* < 0.05. Student's *t*‐test was performed.
**Figure S11:** Increased insulin resistance in PGRMC1 overexpression. (A) Experimental scheme of intramuscular AAV6 injection in PKO mice. (B) Pgrmc1 mRNA expression levels in the liver, adipose tissue and skeletal muscle (Sk.M) of PKO mice compared to the corresponding tissues in WT mice. (C) Blood glucose levels, plasma insulin levels and modified HOMA‐IR in AAV‐injected PKO mice before and after injection. Mice used in experiments: 4 per group. Values represent means ± SD. *, *p* < 0.05. Student's *t*‐test was performed.

**Data S1:** Supporting Information
