# Supplementary material for: Targeting Progesterone Receptor Membrane Component 1 to Improve Muscle Development and Glucose Homeostasis
Source: J Cachexia Sarcopenia Muscle. 2025 Nov 10;16(6):e70121. doi: 10.1002/jcsm.70121 (PMC12598302; doi:10.1002/jcsm.70121)
Supplement: Supplementary file 1 — Figure S1: Generation of Pgrmc1fl/fl mice. sgRNAs targeting Pgrmc1 introns were used to insert loxP sequences, generating the Pgrmc1 floxed allele. Figure S2: Increased AKT phosphorylation in PKO skeletal muscle and P‐KD cells. (A‐B) Western blot analysis and quantification of pmTOR (ser2481), mTOR, phosphorylated mTOR (ser2448), pAKT (ser473) and AKT in WT and PKO skeletal muscles under normal and type 2 diabetes (T2D) conditions. Mice used for the experiment: 4 for normal WT, 4 for normal PKO, 6 for T2D WT and 6 for T2D PKO. (C‐D) Western blot analysis and quantification of PGRMC1, pmTOR (ser2481), mTOR, phosphorylated mTOR (ser2448), pAKT (ser473) and AKT in C‐KD and P‐KD C2C12 or A204 cells. Values represent means ± SD *, p < 0.05. Student's t‐test was performed. For cell culture experiments, n = 4 independent biological replicates were performed. Figure S3: Metabolic phenotype of P‐KD cells. (A) Relative ATP levels in isolated mitochondria from C‐KD and P‐KD A204 cells. ADP (10 μM) was incubated for 1 h at 37°C. (B) Western blot analysis of PGRMC1 and oxidative phosphorylation protein complexes (NDUFB8, CI; SDH8, CII; UQCRC2, CIII; MTCO1, CIV; ATP5A, CV) in C‐KD and P‐KD A204 cells. β‐Actin was used as an internal control. (C) Oxygen consumption rates (OCR) in C‐KD and P‐KD A204 cells were measured using a flux analyser with chemical treatments (oligomycin [Omy]; carbonyl cyanide‐p‐trifluoromethoxyphenylhydrazone [FCCP]; rotenone [Rot]; antimycin [Ant]). Values were normalized to baseline. ATP production rate (OCR change in response to Omy) and maximal respiration rate (OCR change in response to Rot/Ant) were calculated. (D) OCR in C‐KD and P‐KD A204 cells were measured using a flux analyser under the same chemical treatments in media containing BSA‐conjugated palmitate (200 μM). Values were normalized to baseline. ATP production rate (OCR change in response to Omy) and maximal respiration rate (OCR change in response to Rot/Ant) were calculated. Cells were sta [file JCSM-16-e70121-s002.docx]

**Targeting Progesterone Receptor Membrane Component 1 to Improve Muscle development and Glucose Homeostasis.**

**Sang R. Lee^1, 2, 3^, Moeka Mukae^1^, Globinna Kim^4^, Jung-Eun Park^1^, Young Hoon Sung^4^, Young Suk Won^5^, Tae Won Kim^1^, Hyo-Jung Kwun^1^, In-Jeoung Baek^4*^, and Eui-Ju Hong^1*^**

**^1^**College of Veterinary Medicine, Chungnam National University, Daejeon 34134, Republic of Korea

**^2^**Department of Physiology, Dong-A University College of Medicine, Busan 49201, Republic of Korea.

^3^Department of Translational Biomedical Sciences, Graduate School of Dong-A University, Busan, 49201, Republic of Korea.

**^4^**Department of Cell and Genetic Engineering, University of Ulsan College of Medicine, Asan Medical Center, Seoul 05505, Republic of Korea.
**^5^**Laboratory Animal Resource & Research Center, Korea Research Institute of Bioscience and Biotechnology, Cheongju 28116, Republic of Korea.

**^*^**Address correspondence to: In-Jeoung Baek, Department of Cell and Genetic Engineering, University of Ulsan College of Medicine, Asan Medical Center, Seoul 05505, Korea. Phone: +82-2-3010-2798; Fax: +82-2-3010-4197 Email: ijbaek@amc.seoul.kr

**^*^**Address correspondence to: Eui-ju Hong, College of Veterinary Medicine, 99Daehak-ro, Suite 401Veterinary medicine Bldg., Yuseong, Daejeon 34134, Korea. Phone: +82-42-821-6781;Fax: +82-42-821-8903 Email: [ejhong@cnu.ac.kr](mailto:ejhong@cnu.ac.kr)


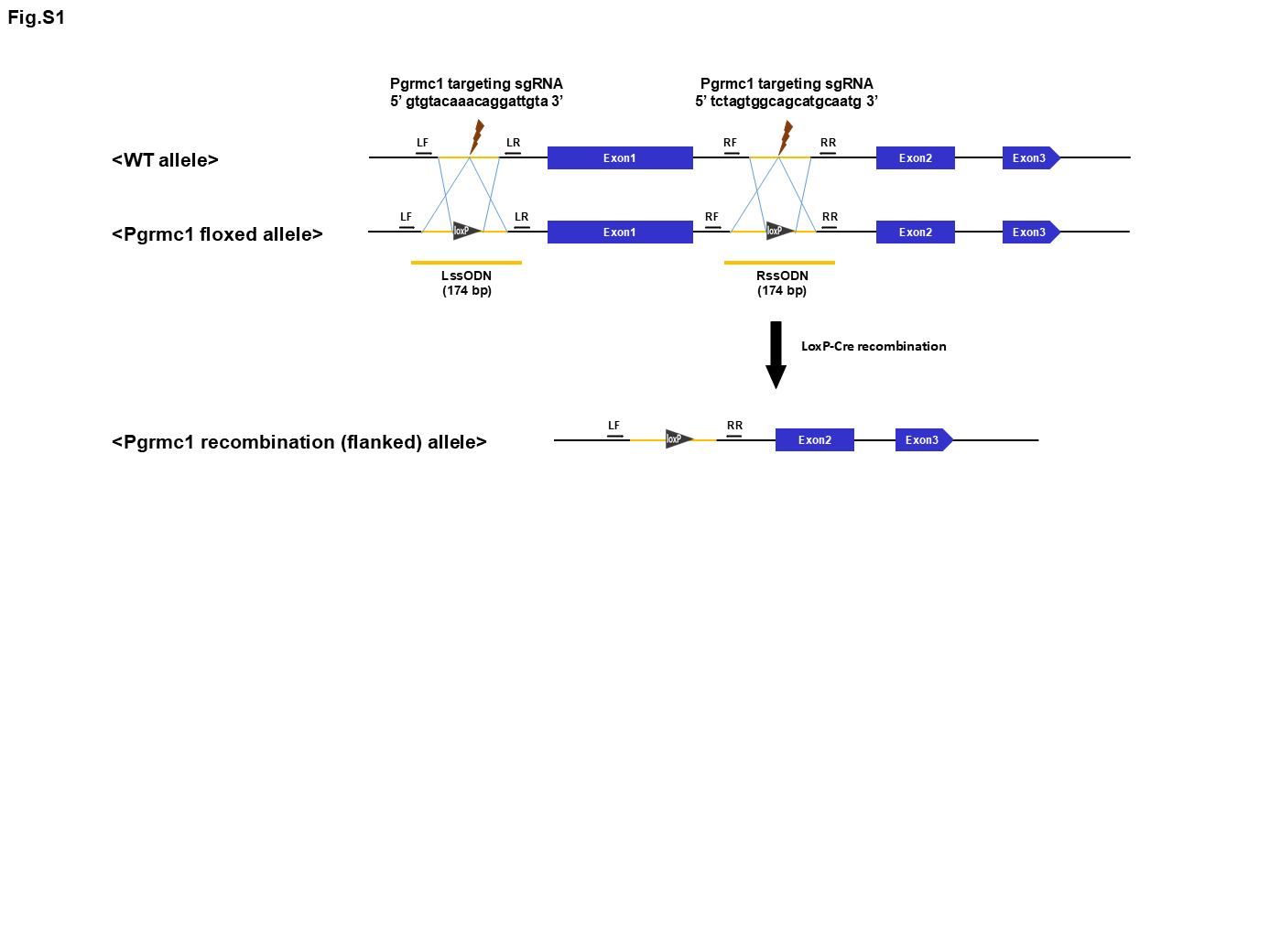


**Figure S1.** Generation of Pgrmc1^fl/fl^ mice. sgRNAs targeting Pgrmc1 introns were used to insert loxP sequences, generating the Pgrmc1 floxed allele.


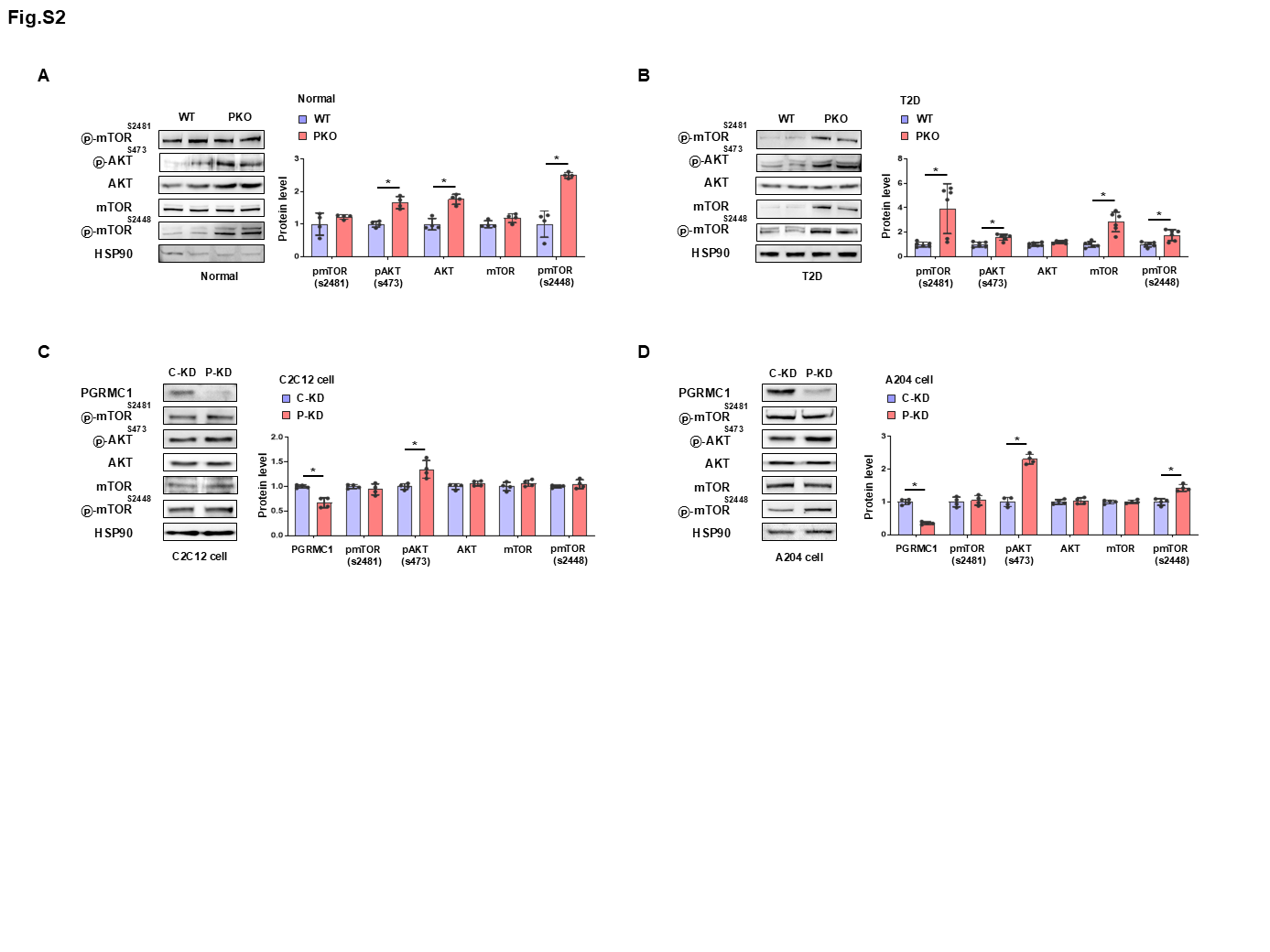


**Figure S2.** Increased AKT phosphorylation in PKO skeletal muscle and P-KD cells. (A-B) Western blot analysis and quantification of pmTOR (ser2481), mTOR, phosphorylated mTOR (ser2448), pAKT (ser473), and AKT in WT and PKO skeletal muscles under normal and type 2 diabetes (T2D) conditions. Mice used for the experiment:4 for normal WT, 4 for normal PKO, 6 for T2D WT, and 6 for T2D PKO. (C-D) Western blot analysis and quantification of PGRMC1, pmTOR (ser2481), mTOR, phosphorylated mTOR (ser2448), pAKT (ser473), and AKT in C-KD and P-KD C2C12 or A204 cells. Values represent means ± SD. *, p<0.05. Student’s t-test was performed. For cell culture experiments, n = 4 independent biological replicates were performed.

**
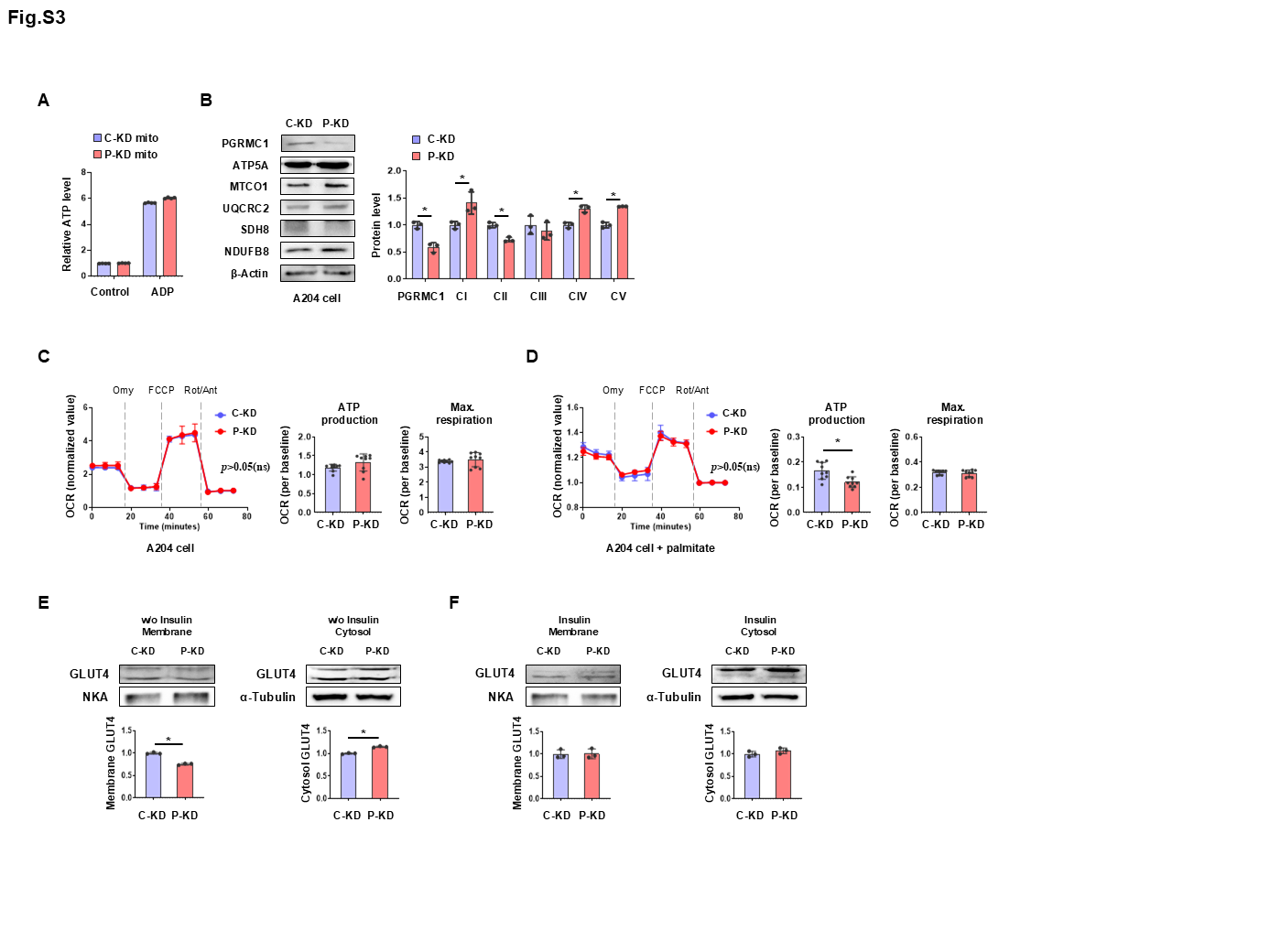
**

**Figure S3.** Metabolic phenotype of P-KD cells. (A) Relative ATP levels in isolated mitochondria from C-KD and P-KD A204 cells. ADP (10 μM) was incubated for 1 hour at 37°C. (B) Western blot analysis of PGRMC1 and oxidative phosphorylation protein complexes (NDUFB8, CI; SDH8, CII; UQCRC2, CIII; MTCO1, CIV; ATP5A, CV) in C-KD and P-KD A204 cells. β-Actin was used as an internal control. (C) Oxygen consumption rates (OCR) in C-KD and P-KD A204 cells were measured using a flux analyzer with chemical treatments (oligomycin [Omy]; carbonyl cyanide-p-trifluoromethoxyphenylhydrazone [FCCP]; rotenone [Rot]; antimycin [Ant]). Values were normalized to baseline. ATP production rate (OCR change in response to Omy) and maximal respiration rate (OCR change in response to Rot/Ant) were calculated. (D) OCR in C-KD and P-KD A204 cells were measured using a flux analyzer under the same chemical treatments in media containing BSA-conjugated palmitate (200 μM). Values were normalized to baseline. ATP production rate (OCR change in response to Omy) and maximal respiration rate (OCR change in response to Rot/Ant) were calculated. Cells were starved for 5 hours and treated with insulin (100 nM) for 30 minutes. (E-F) Western blot analysis and quantification of GLUT4 in A204 cells. Cells were either untreated or treated with insulin (100 nM). α-Tubulin or NKA was used as an internal control. Values represent mean ± SD. *p < 0.05, Student’s t-test and two-way ANOVA (column factor) test were performed. For cell culture experiments: Panel A, n = 4 independent biological replicates; Panel B-F, n = 3 independent biological replicates.

**
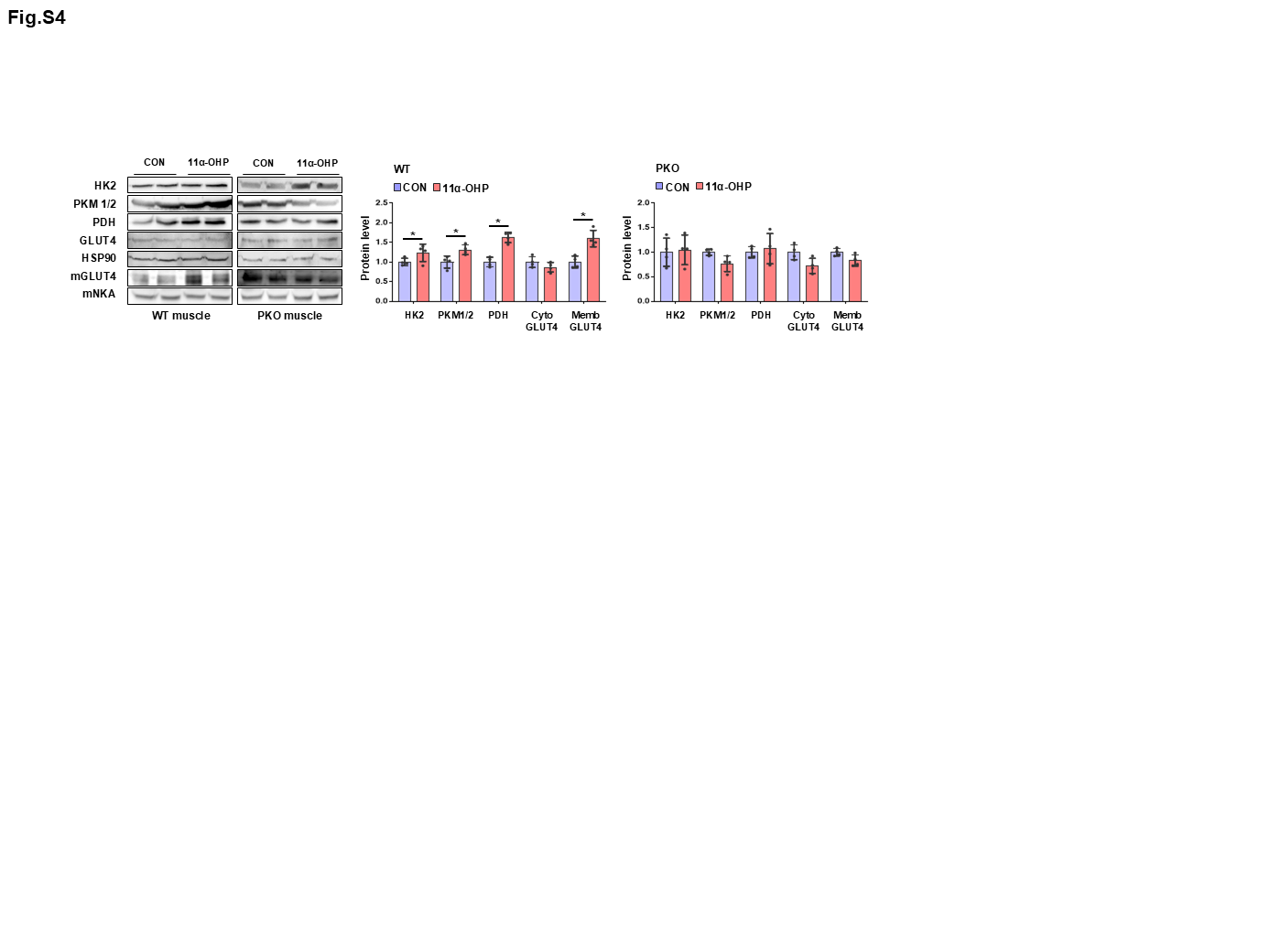
**

**Figure S4.** Increased glycolytic enzyme and glucose uptake protein levels in skeletal muscles of 11α-OHP-treated mice only in the presence of PGRMC1. Western blot analysis and quantification of glycolytic enzymes (HK2, PKM1/2, and PDH) and the glucose transporter GLUT4 in skeletal muscles of WT and PKO mice treated with 11α-OHP. Mice used for experiments: 4 for each group. HSP90 and NKA were used as internal controls. Values represent means ± SD. *, p<0.05. Student’s t-test was performed.


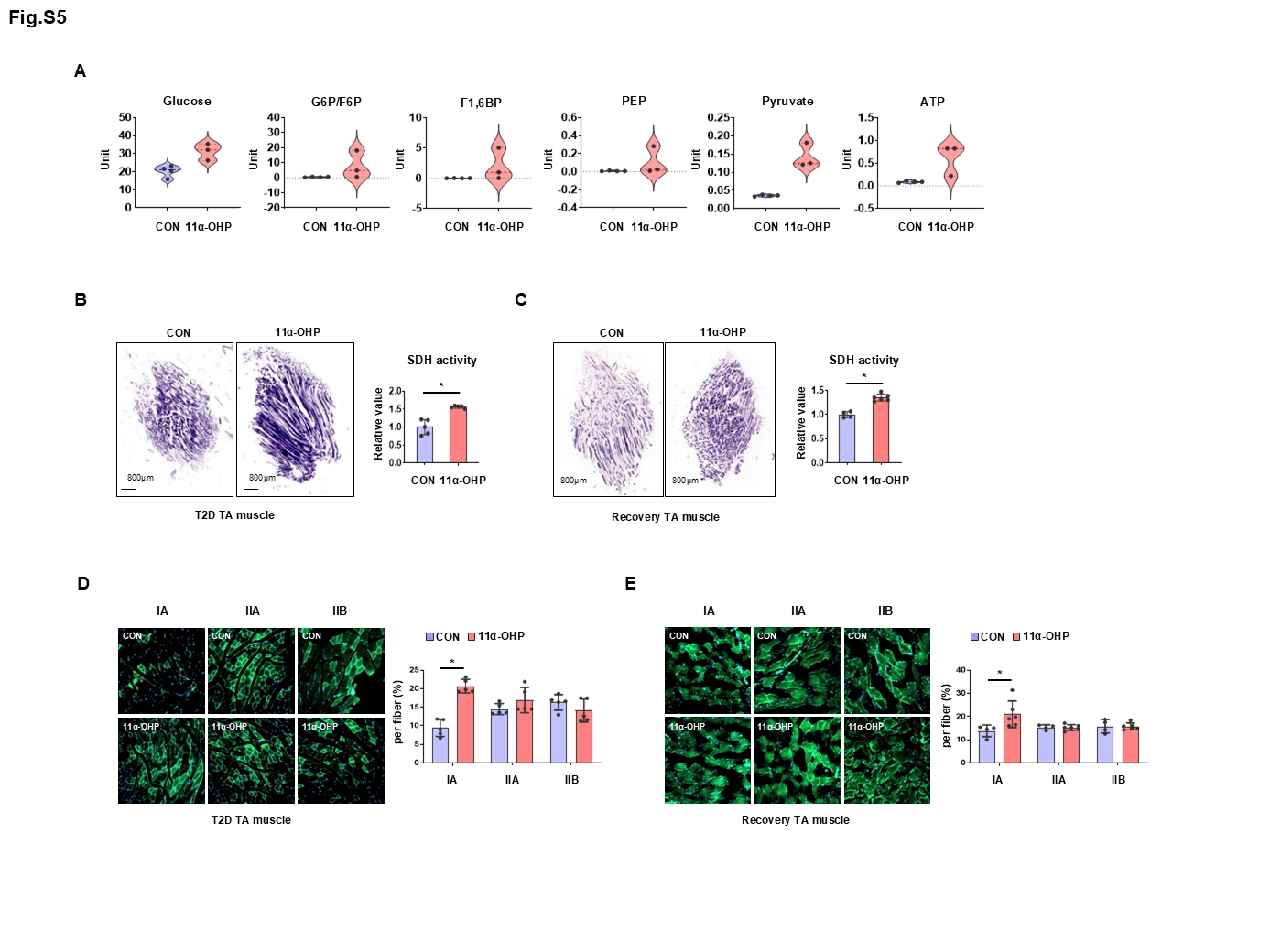


**Figure S5.** Increased glycolytic metabolites, mitochondrial metabolism, and oxidative fiber abundance in 11α-OHP-treated skeletal muscle. (A) Quantification of glucose and glucose metabolites in WT and PKO skeletal muscles using LC/MS. The unit represents the relative ratio, calculated as the analyte peak area divided by the internal standard peak area and normalized to tissue weight (mg). (B-C) SDH staining of skeletal muscle from vehicle-treated and 11α-OHP-treated mice in both the developing and recovery stages of T2D. (D-E) Immunostaining of type IA, IIA, and IIB muscle fibers in skeletal muscle from vehicle-treated and 11α-OHP-treated mice in both the developing and recovery stages of T2D. Mice used in the experiment: 5 for T2D-developing control, 5 for T2D-developing 11α-OHP, 4 for T2D-recovering control, and 6 for T2D-recovering 11α-OHP. Values represent means ± SD. *, p<0.05. Student’s t-test was performed.

**
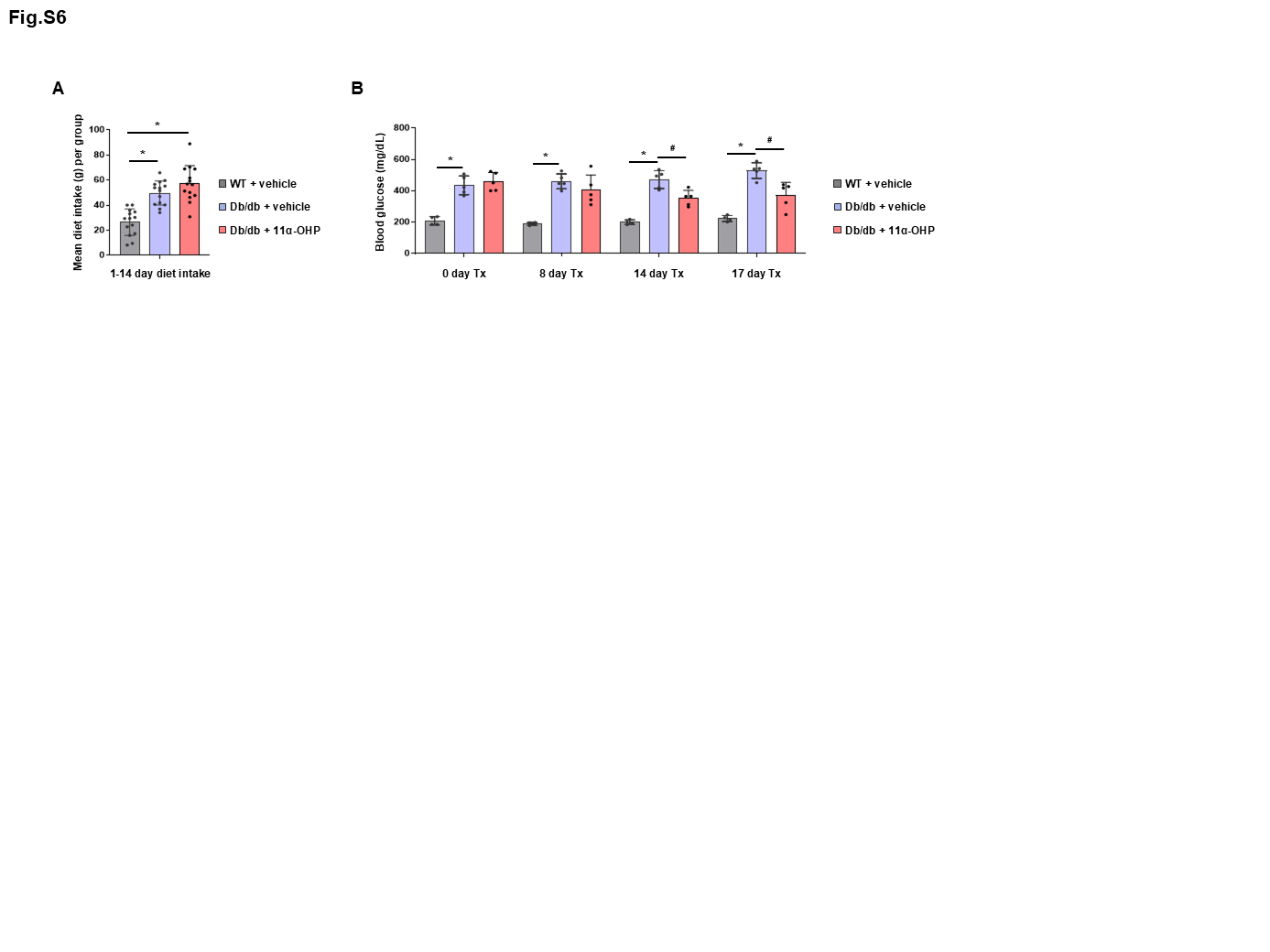
**

**Fig. S6.** Monitoring of diet intake and blood glucose levels of Db/db mice. (A) Average diet intake (g) per group in Db/db mice. Diet intake was measured collectively for each group, not individually. (B) Blood glucose level monitoring throughout the treatment period. Values represent means ± SD. *, p<0.05. Mice used for the experiment:4 for control, 5 for *db/db* control, and 5 for *db/db* 11α-OHP. One-way ANOVA followed by a tukey’s multiple comparisons test were performed.


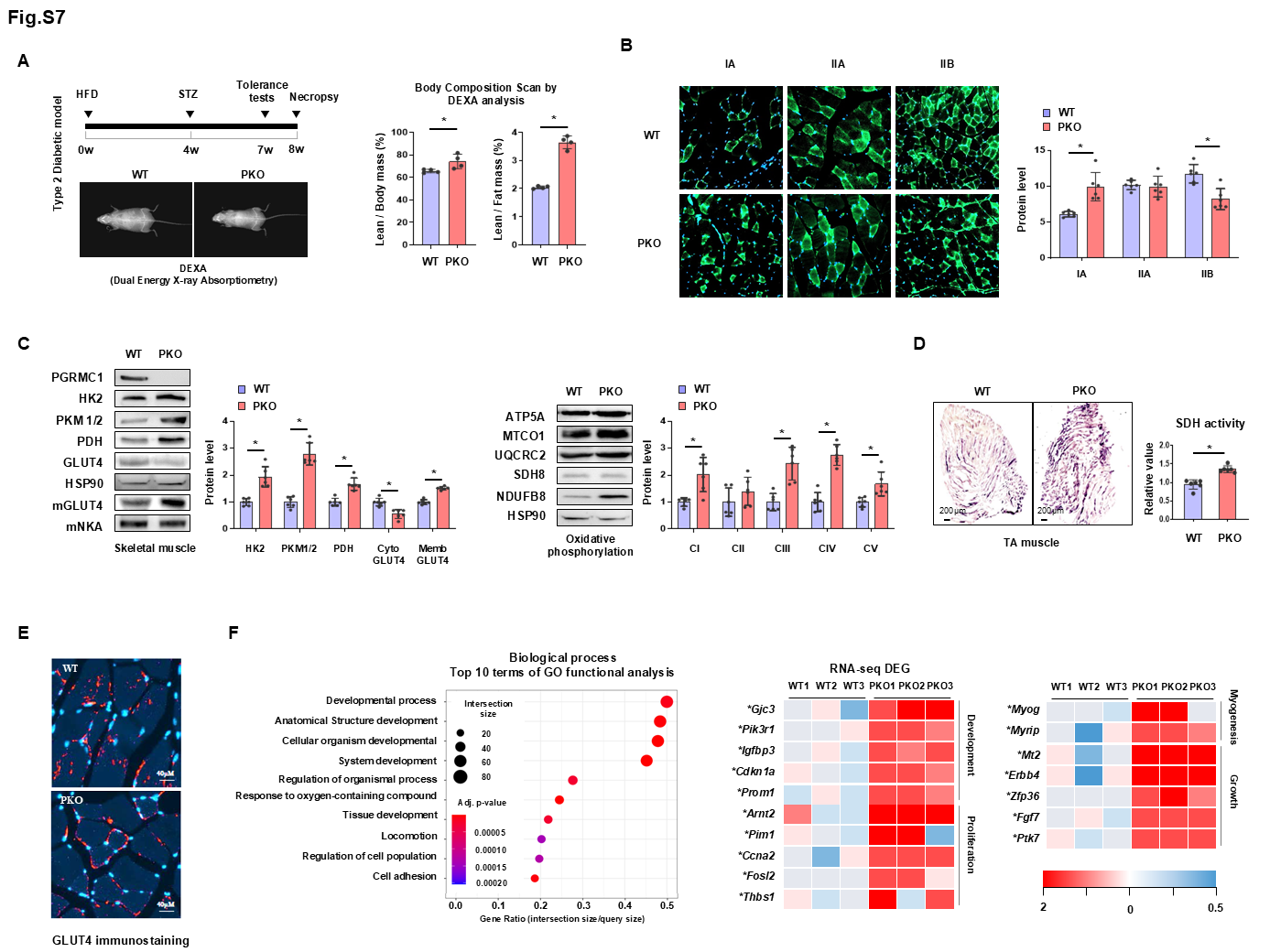
**Figure S7.** Systemic PKO retains metabolic activation of skeletal muscle. (A) Experimental schedule for type 2 diabetes (T2D) induction and representative dual X-ray absorptiometry (DEXA) images of T2D WT and PKO mice. Streptozotocin (STZ, 30 mg/kg) was administered intraperitoneally. DEXA measurements showing lean mass relative to body mass (%) and fat mass (%) in WT and PKO mice. Mice used for the analysis: 4 per group. (B) Immunostaining of type IA, IIA, and IIB muscle fibers in WT and PKO skeletal muscles. (C) Western blot analysis and quantification of key enzymes involved in glycolysis and glucose uptake in WT and PKO skeletal muscles. HSP90 and membrane NKA were used as internal controls. Western blot analysis and quantification of key enzymes involved in oxidative phosphorylation in WT and PKO skeletal muscles. HSP90 was used as internal control. (D) SDH staining of the tibialis anterior (TA) skeletal muscle in WT and PKO mice. SDH enzymes are visualized in purple. Mice used for the experiment: 6 per group. (E) Immunostaining of GLUT4 (red) in WT and PKO skeletal muscles. DAPI (blue) was used as a nuclear counterstain. (F) Top 10 biological processes showing significant differences in PKO skeletal muscle based on RNA sequencing analysis. Heatmap of differentially expressed mRNAs related to development, proliferation, myogenesis, and growth in WT and PKO skeletal muscle from RNA sequencing analysis. Fold changes are indicated by color (red, increase; blue, decrease). Mice used for RNA sequencing analysis: 3 per group. Values represent means ± SD. *, p<0.05. Student’s t-test was performed.

**
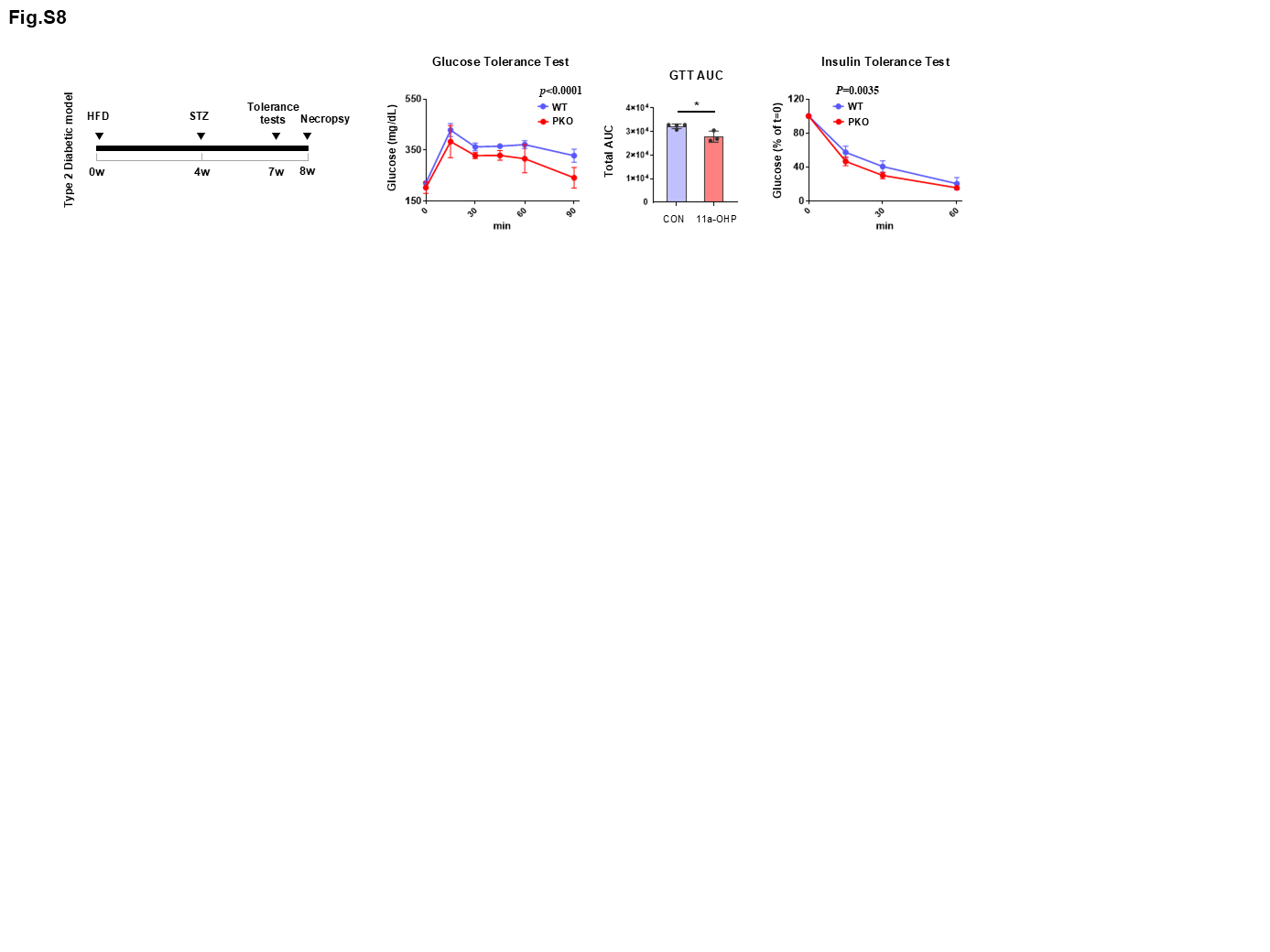
**

**Figure S8.** Systemic PKO improves glucose clearance and insulin responsiveness in T2D. Experimental schedule for type 2 diabetes (T2D) induction. Streptozotocin (STZ, 30 mg/kg) was administered intraperitoneally. Glucose tolerance test (GTT) and insulin tolerance test (ITT) in WT and PKO mice. Mice used in experiments: 4 (WT) and 3 (PKO). Values represent mean ± SD. *p < 0.05, Student’s t-test. Two-way ANOVA (column factor) was performed for GTT and ITT.

**
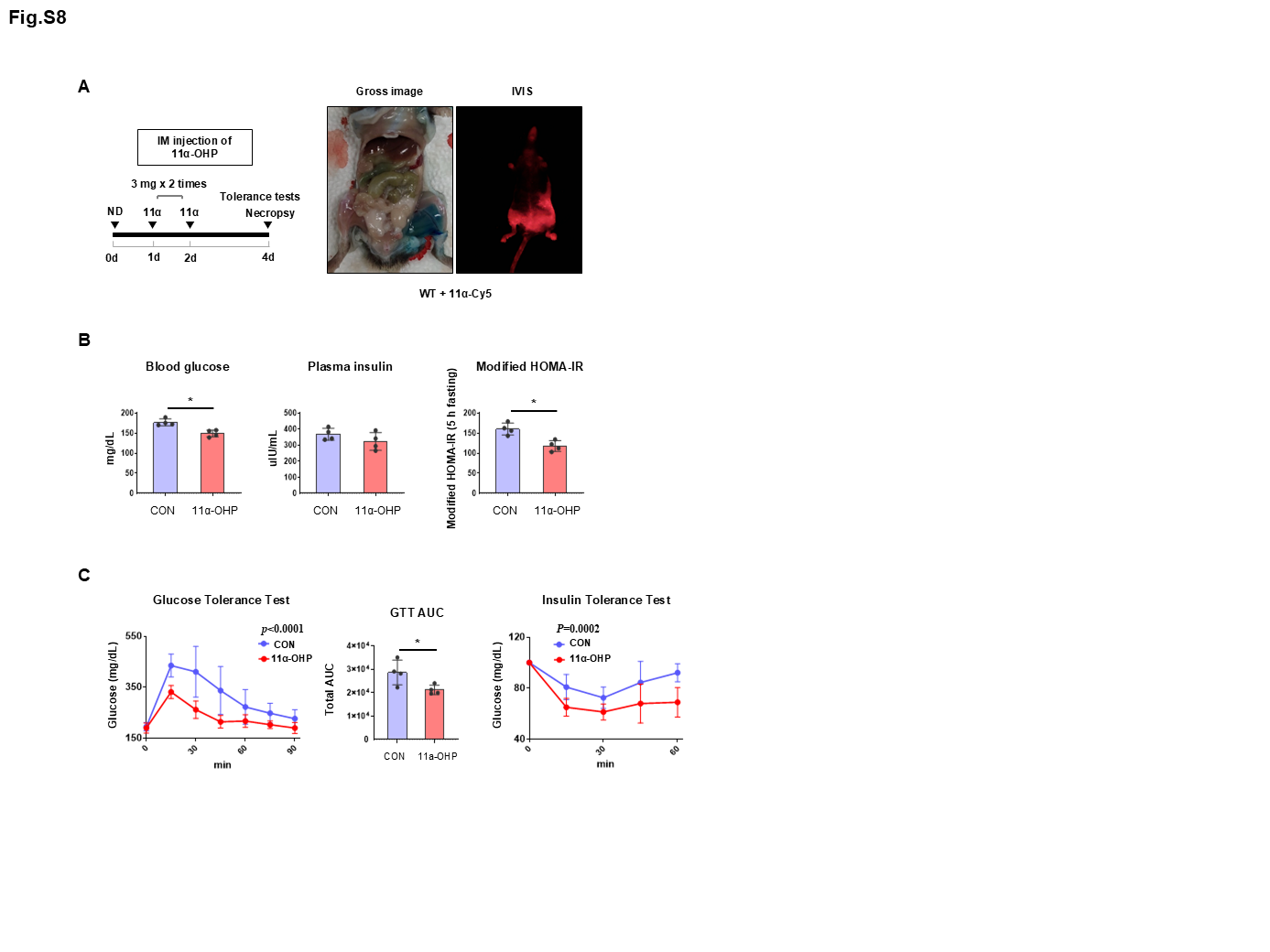
**

**Fig. S9.** Enhanced glucose clearance and insulin sensitivity by intramuscular injection of 11α-OHP. (A) Schematic representation of the intramuscular injection of 11α-OHP. Gross image of sacrificed mice displaying Cy5-labeled 11α-OHP as a blue tint. In vivo imaging showing the distribution of Cy5-labeled 11α-OHP. (B) Blood glucose levels, plasma insulin levels, and modified HOMA-IR in vehicle-treated and 11α-OHP-treated mice. (C) Glucose tolerance test (GTT) and insulin tolerance test (ITT) in vehicle-treated and 11α-OHP-treated mice. Mice used in experiments: 4 per group. Values represent mean ± SD. *p < 0.05, Student’s t-test. Two-way ANOVA (column factor) was performed for GTT and ITT.


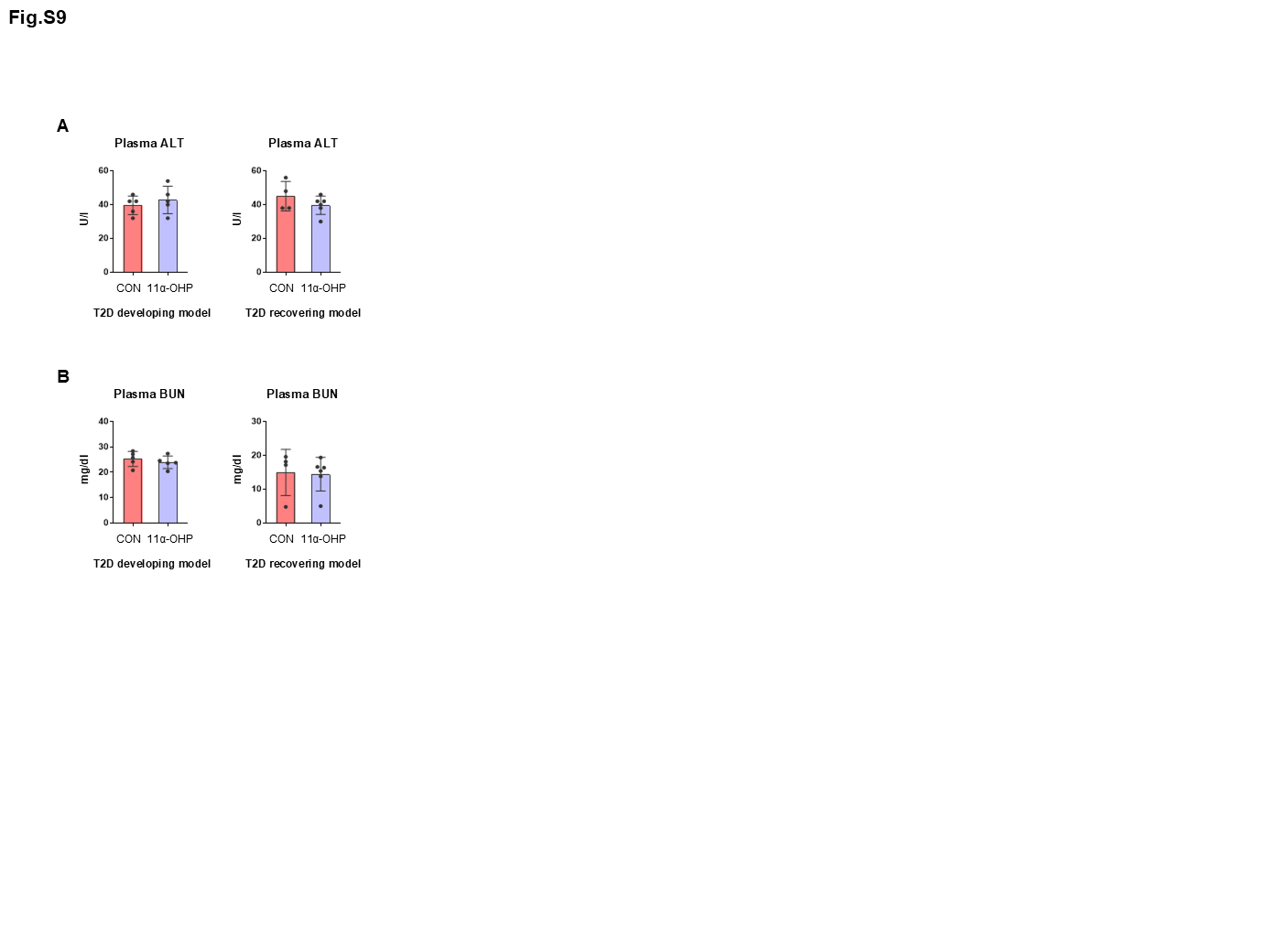


**Fig. S10.** Blood markers of hepatotoxicity and nephrotoxicity in 11α-OHP treated mice. (A) Plasma ALT levels of vehicle-treated and 11α-OHP-treated mice in both the developing and recovery stages of T2D. (B) Plasma BUN levels of vehicle-treated and 11α-OHP-treated mice in both the developing and recovery stages of T2D. Mice used in the experiment: 5 for T2D-developing control, 5 for T2D-developing 11α-OHP, 4 for T2D-recovering control, and 6 for T2D-recovering 11α-OHP. Values represent means ± SD. *, p<0.05. Student’s t-test was performed.


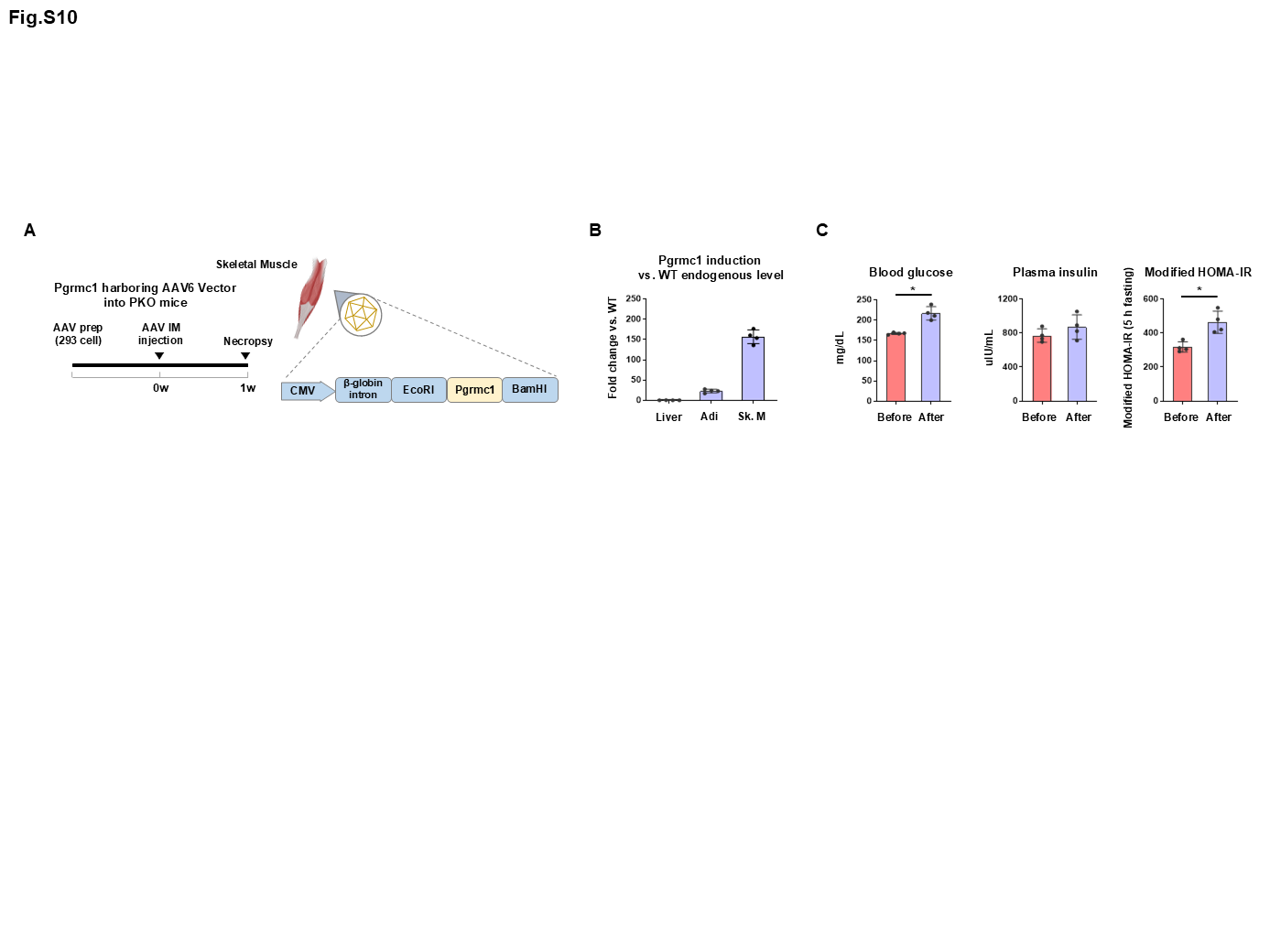


**Fig. S11.** Increased insulin resistance in PGRMC1 overexpression. (A) Experimental scheme of intramuscular AAV6 injection in PKO mice. (B) Pgrmc1 mRNA expression levels in the liver, adipose tissue, and skeletal muscle (Sk.M) of PKO mice compared to the corresponding tissues in WT mice. (C) Blood glucose levels, plasma insulin levels, and modified HOMA-IR in AAV-injected PKO mice before and after injection. Mice used in experiments: 4 per group. Values represent means ± SD. *, p<0.05. Student’s t-test was performed.
