# Supplementary material for: Targeting Progesterone Receptor Membrane Component 1 to Improve Muscle Development and Glucose Homeostasis
Source: J Cachexia Sarcopenia Muscle. 2025 Nov 10;16(6):e70121. doi: 10.1002/jcsm.70121 (PMC12598302; doi:10.1002/jcsm.70121)
Supplement: Supplementary file 2 — Data S1: Supporting Information [file JCSM-16-e70121-s001.docx]

**Targeting Progesterone Receptor Membrane Component 1 to Improve Muscle development and Glucose Homeostasis.**

**Sang R. Lee^1, 2, 3^, Moeka Mukae^1^, Globinna Kim^4^, Jung-Eun Park^1^, Young Hoon Sung^4^, Young Suk Won^5^, Tae Won Kim^1^, Hyo-Jung Kwun^1^, In-Jeoung Baek^4*^, and Eui-Ju Hong^1*^**

**^1^**College of Veterinary Medicine, Chungnam National University, Daejeon 34134, Republic of Korea

**^2^**Department of Physiology, Dong-A University College of Medicine, Busan 49201, Republic of Korea.

^3^Department of Translational Biomedical Sciences, Graduate School of Dong-A University, Busan, 49201, Republic of Korea.

**^4^**Department of Cell and Genetic Engineering, University of Ulsan College of Medicine, Asan Medical Center, Seoul 05505, Republic of Korea.
**^5^**Laboratory Animal Resource & Research Center, Korea Research Institute of Bioscience and Biotechnology, Cheongju 28116, Republic of Korea.

**^*^**Address correspondence to: In-Jeoung Baek, Department of Cell and Genetic Engineering, University of Ulsan College of Medicine, Asan Medical Center, Seoul 05505, Korea. Phone: +82-2-3010-2798; Fax: +82-2-3010-4197 Email: ijbaek@amc.seoul.kr

**^*^**Address correspondence to: Eui-ju Hong, College of Veterinary Medicine, 99Daehak-ro, Suite 401Veterinary medicine Bldg., Yuseong, Daejeon 34134, Korea. Phone: +82-42-821-6781;Fax: +82-42-821-8903 Email: [ejhong@cnu.ac.kr](mailto:ejhong@cnu.ac.kr)

**2.1. Generation of skeletal muscle-specific *Pgrmc1* KO mice**

To generate single guide RNAs (sgRNAs) for floxed *Pgrmc1* mice, the following oligomers were purchased from Macrogen, Inc.: 5′−taggGTGTACAAACAGGATTGTA−3′ and 5′−aaacTACAATCCTGTTTGTACAC−3′ for *Pgrmc1* 5’UTR-specific sgRNA and 5′−aaacTCTAGTGGCAGCATGCAATG−3′ and 5′−aaac CATTGCATGCTGCCACTAGA−3′ for *Pgrmc1* intron 1-specific sgRNA. Each pair of oligomers was annealed and cloned into the pUC57-sgRNA vector, a gift from Xingxu Huang (Addgene plasmid # 51132; <http://n2t.net/addgene:51132;RRID:Addgene_51132>), using BsaI restriction enzymes (New England BioLabs). The cloned vectors were then used as templates for sgRNA in vitro transcription. The following single-stranded deoxynucleotide donor DNAs were purchased from Integrated DNA Technologies, Inc.: 5′−caaaagttttcagaatttagccaattctctgcacaaatctcttgcatgtaagtggtgtacaaacaggattATAACTTCGTATAATGTATGCTATACGAAGTTATgtatggaaaagtcgtgccctgctctgcagatgtgtgcatggcttggttttaattattctcctttttctta−3′ for inserting a loxP into *Pgrmc1* 5’UTR and 5′−tagcagtatatattttgtgaaaagcatatgaattttgttctttttaaaccttctctagtggcagcatgcaATAACTTCGTATAATGTATGCTATACGAAGTTATatgaggactgaaaccaacccctattttagaatcttagttgcatttacaagatgatcctaagggtttgaag−3′ for inserting a loxP into *Pgrmc1* intron 1. Microinjection of fertilized eggs and their subsequent transfer to foster mothers were performed as previously described [1]. Genomic DNA samples extracted from the tails of 3-week-old pups were used for genotyping.

For the skeletal muscle-specific Pgrmc1 knockout model, ACTA-cre/Esr1* transgenic mice (Jackson Laboratory, Stock #025750) were obtained from Jackson Laboratory. The skeletal muscle-specific Pgrmc1 knockout (ACTAcre-Pgrmc1^fl/fl^) was generated by breeding Pgrmc1^fl/fl^ mice with mice expressing Cre recombinase under the control of the actin alpha-1, skeletal muscle promoter. ACTAcre-Pgrmc1^fl/fl^ mice were administered intraperitoneal injections of tamoxifen (2 mg per 20–22 g body weight) daily for 5 consecutive days to induce cre-mediated deletion.

**2.2. Chemicals**

The following chemicals were used in this study: insulin (Welgene, ls038-01; in vitro), glucose (Welgene, ls001-02; in vitro), glucose (Sigma, G8270; in vivo), pyruvate (Sigma, P2256), insulin (Sigma, 91077C; in vivo), 11α-hydroxyprogesterone (Santa Cruz, sc-287291), rapamycin (TCI, R0097), alpelisib (Selleckchem, S2814), ipatasertib (Selleckchem, S2808), BI-D1870 (Selleckchem, S2843), and okadaic acid (MedChem Express, HY-N6785).

**2.3. Blood glucose level, tolerance tests, and HOMA-IR**

Blood glucose level was measured using the Accu-Chek Active kit (Roche, 07124112) after the indicated fasting period. To minimize sympathetic disturbances, mice were not restrained before tail snipping. For the glucose tolerance test (GTT) and insulin tolerance test (ITT), glucose (2 g/kg) and insulin (0.75 U/kg) were administered intraperitoneally, respectively, after a 5-hour fasting period. Blood glucose levels were measured at the specified time points. The fasting time points of blood glucose and insulin measurements varied depending on the experimental conditions. Modified HOMA-IR was recorded when HOMA-IR was measured after a 5-hour fasting period required for resting state induction before necropsy. The homeostatic model assessment of insulin resistance (HOMA-IR) was calculated using the equation: HOMA-IR = (blood glucose (mg/dL) x insulin level (uIU/mL) / 405).

**2.4. Western Blotting**

Protein samples were extracted through a sequential process of homogenization, centrifugation, and sonication from tissues and cells. Protein concentrations were measured using the PRO-Measure solution (Intron, #21011) and subjected to SDS-PAGE. The separated proteins were transferred onto PVDF membranes (Merk Millipore, IPVH00010). The membranes were then blocked with 3 % BSA and incubated with primary antibodies overnight. On the following day, the membranes were incubated with the appropriate secondary antibodies, and protein signals were detected using an ECL solution (XLS025-0000, Cyanagen) and a ChemiDoc imaging system (Fusion Solo, Vilber Lourmat).

Primary antibodies: HSP90 (Cell Signaling Technology; CST, #4874), Na+/K+-ATPase (NKA; Abclonal, A11683), total OXPHOS (Abcam, ab110413), pAKT (ser473) (CST, #4060), AKT (CST, #9272), GLUT4 (Abcam, ab654), Glycolysis Antibody Sampler Kit (CST, #8337), Insulin/IGF-1 Signaling Pathway Antibody Sampler Kit (CST, #42022), PGRMC1 (CST, #13856), mTOR Pathway Antibody Sampler Kit (CST, #9964), pRSK1 (Abclonal, AP0767), RSK1 (Abclonal, A4695), PPP2R5D (Abclonal, A21122) β-Actin (Santa Cruz, sc-47778), α-Tubulin (66031-1-Ig, Proteintech).

Secondary antibodies: Goat anti-Rabbit IgG HRP (Thermo Fisher Scientific, 31460), Goat anti-Mouse IgG HRP (Thermo Fisher Scientific, 31430).

**2.5. Oxidative capacity and ATP production measurements**

To assess oxidative capacity via succinate dehydrogenase (SDH) activity, tibialis anterior (TA) skeletal muscle samples were frozen, sectioned using a cryostat, and dried for 1 hour. The sections were then incubated for 1 hour at 37 ℃ in a solution containing 200 mM phosphate buffer, 100 mM MgCl_2_, 200 mM succinic acid, and 2.4 mM nitroblue tetrazolium. The entire skeletal muscle section was observed under a light microscope to evaluate SDH activity. For ATP production measurement, mitochondria were isolated from A204 cell based on previous protocol [2] and processed using a commercial ATP assay kit (Abcam, ab65313, Dogenbio, DG-ATP100) following the manufacturer’s protocol.

**2.6. Mitochondrial respiration, glycolysis, and fatty acids oxidation measurements**

A204 cells were cultured in a Seahorse cell culture plate and starved in McCoy’s medium (4500 mg/L glucose, without FBS) for 5 hours to remove endogenous hormones. For glycolysis measurements, cells were incubated in low-glucose McCoy’s medium (500 mg/L glucose, without FBS) for 1 hour. Cells were then decarboxylated for 40 minutes to 1 hour in Seahorse XFp medium (103575-100, Agilent technologies), supplemented with the same concentrations of glutamine, sodium pyruvate, and glucose as the medium in which cells were grown. For the mitochondrial stress test, cells were sequentially treated with oligomycin (2 µM), FCCP (0.5 µM), and rotenone/antimycin (0.5 µM). Oxygen consumption rate (OCR) was measured following treatment. For the fatty acid oxidation test, BSA-conjugated palmitate (200 µM) was added. For the glycolysis stress test, cells were treated with glucose (25 mM) and oligomycin (2 µM), and extracellular acidification rate (ECAR) was measured. Prior to the stress tests, insulin (100 nM) was administered for 20 minutes. All measurements were performed using the Seahorse XFp analyzer (Agilent technologies) with the Seahorse XFp FluxPak (103022-100, Agilent technologies).

**2.7. Immunostaining**

Tissue sections were cut from paraffin-embedded block, attached to slides, and warmed in a dry oven. The slides were deparaffinized in xylene and subjected to a series of rehydration steps (100% ethanol, 90% ethanol, 70% ethanol, and distilled water). Antigen retrieval was performed by incubating the slides in sodium citrate buffer at 95℃. Alternatively, frozen sections were obtained using a cryostat, dried, and washed with TBS-T. After blocking with 3% BSA, slides were incubated overnight at 4°C with primary antibodies. Following washes with TBS-T, secondary antibodies were incubated overnight at 4°C. DAPI (Life Technologies, 1674645) was applied for nuclear staining. Primary antibodies: MHC-IA (DSHB, BA-D5), MHC-IIA (DSHB, 2F7), MHC-IIB (DSHB, 10F5), and GLUT4 (Abcam, ab654). Secondary antibodies: anti-rabbit (A21207, Life Technologies) and anti-mouse (A21202, Life Technologies) antibodies.

**2.8. RNA sequencing**

The transcriptomes of WT and PKO muscle samples were sequenced using next-generation sequencing (NGS) by Macrogen. For quality control, genes with a count of zero in more than one sample were excluded from analysis. As a result, 15,642 genes were retained for statistical analysis, while 30,135 genes were excluded.

**2.9. Cell Culture**

All cell culture reagents were purchased from Welgene (Gyeongsan, Korea). A204 human rhabdomyosarcoma cells were maintained at 37°C in a 5% CO₂ atmosphere in McCoy's medium (Welgene, LM005-01) supplemented with 5% (vol/vol) fetal bovine serum (FBS), penicillin (100 U/mL), and streptomycin (100 μg/mL). C2C12 mouse myoblasts were cultured under the same conditions in DMEM medium (Welgene, LM001-05) supplemented with 5% FBS, penicillin (100 U/mL) and streptomycin (100 μg/mL). For PGRMC1 (siRNA sequence: CAGUACAGUC GCUAGUCAA, CAGUUCACUU UCAAGUAUCA U) and PPP2R5D (GAAGGGCAGG UUCCGAAUGA A) knockdown, cells were transfected using Lipofectamine 2000 (Thermo Fisher, 11668-027) following the manufacturer’s protocol. For C2C12 myoblast differentiation, cells were incubated in differentiation medium containing 0.2 % FBS for 4 days, and differentiation was monitored by microscope. To assess differentiation index, anti-MyHC (DSHB, MF20) was immunostained and the MyHC^+^ nuclei per total nuclei (DAPI) was measured by Image J. To measure the PP2A activity, cells were processed to PP2A immunoprecipitation phosphatase assay kit (Sigma-Aldrich, 17-313) according to manufacturer’s protocol.

**2.10. Crude membrane protein separation**

Membrane proteins were extracted following a previously established protocol [3]. Samples were homogenized in buffer containing 0.25 M sucrose, 20 mM Tris-HCl, and 1 mM EDTA (pH 7.5). Supernatants were collected by centrifugation at 1,000 × g for 10 minutes, followed by an additional centrifugation at 14,000 × g for 20 minutes. The resulting pellets were lysed in T-PER buffer and sonicated for further analysis.

**2.11. 11α-OHP screening process**

A total of 330 chemicals with structural relationships to Pgrmc1 or sigma-2 receptor modulators were screened for their ability to reduce Pgrmc1 protein levels at a single concentration (100 nM). Candidate compounds identified as Pgrmc1 suppressors were further tested at multiple doses to assess dose-dependent responses. Additionally, effector responses such as AKT phosphorylation and glycolysis rate (Seahorse assay) were evaluated to determine Pgrmc1-mediated effects. After these screening steps, only 11α-OHP met the criteria for further in vivo testing in mice.

**2.12. 11α-OHP concentration in blood**

A stock solution of 11α‑OHP was prepared in ethanol and stored at 4 °C, and calibration standards (0–1,000 ng/mL) were generated by serial dilution of this stock with ethanol. Quality control (QC) samples and calibration standards were prepared by spiking these working solutions into blank mouse serum at designated concentrations. For sample preparation, 150 μL of mouse serum was mixed with 20 μL methanol and 20 μL of 1 M ammonium hydroxide, followed by vortexing for 30 s and liquid–liquid extraction with 600 μL methyl tert-butyl ether (MTBE) for 3 min. After centrifugation at 4,000 rpm (1,163 × g) for 5 min, 500 μL of the organic layer was collected, evaporated to dryness under nitrogen at ~30 °C, and reconstituted in 100 μL methanol. The sample was vortexed for 1 min, centrifuged at 12,000 rpm (10,464 × g) for 5 min, and 80 μL of the supernatant was injected into an Agilent UPLC-6470TQ LC–MS/MS system equipped with an Eclipse Plus C18 column (2.1 × 100 mm, 3.5 μm) maintained at 40 °C. Chromatographic separation was achieved isocratically with 0.1% formic acid in water and acetonitrile (50:50, v/v) at a flow rate of 0.300 mL/min for 6 min. Mass spectrometric detection was performed in positive electrospray ionization (ESI+) mode using multiple reaction monitoring (MRM), with the precursor ion m/z 331 and product ions m/z 331→109 (quantitative, 30 eV) and m/z 331→97.1 (confirmatory, 35 eV). Fragmentor voltage was set at 95 V, dwell time at 200 ms, and cell accelerator voltage at 5 V.

**References**

1. Sung YH, Baek IJ, Kim DH, Jeon J, Lee J, Lee K, et al. Knockout mice created by TALEN-mediated gene targeting. Nature biotechnology. 2013;31:23-4.

2. Liao PC, Bergamini C, Fato R, Pon LA, Pallotti F. Isolation of mitochondria from cells and tissues. Methods Cell Biol. 2020;155:3-31.

3. Du K, Murakami S, Sun Y, Kilpatrick CL, Luscher B. DHHC7 Palmitoylates Glucose Transporter 4 (Glut4) and Regulates Glut4 Membrane Translocation. J Biol Chem. 2017;292:2979-91.
